# The Iron Deficiency-Regulated Small Protein Effector FEP3/IRON MAN1 Modulates Interaction of BRUTUS-LIKE1 With bHLH Subgroup IVc and POPEYE Transcription Factors

**DOI:** 10.3389/fpls.2022.930049

**Published:** 2022-06-10

**Authors:** Daniela M. Lichtblau, Birte Schwarz, Dibin Baby, Christopher Endres, Christin Sieberg, Petra Bauer

**Affiliations:** ^1^Institute of Botany, Heinrich Heine University Düsseldorf, Düsseldorf, Germany; ^2^Cluster of Excellence on Plant Sciences, Heinrich Heine University Düsseldorf, Düsseldorf, Germany

**Keywords:** Fe deficiency, protein interaction network, bHLH, yeast three-hybrid, tripartite interaction, BRUTUS-LIKE E3 ligase, *Arabidopsis thaliana*, IRON MAN

## Abstract

In light of climate change and human population growth one of the most challenging tasks is to generate plants that are Fe-efficient, resilient to low Fe supply and Fe-biofortified. For such endeavors, it is crucial to understand the regulation of Fe acquisition and allocation in plants. One open question is how identified Fe-regulatory proteins comprising positive and negative regulators act together to steer Fe homeostasis. bHLH transcription factors (TFs) belonging to the subgroups IVb and IVc can initiate a bHLH cascade controlling the –Fe response in roots. In *Arabidopsis thaliana*, the –Fe-induced genes are sub-divided into several gene co-expression clusters controlled by different sets of TFs. Some of the co-expressed genes encode regulatory E3 ligase proteins BRUTUS (BTS)/BTS-LIKE (BTSL) and small proteins belonging to the group of FE UPTAKE-INDUCING PEPTIDE/IRON MAN (FEP/IMA). Recently, it was described that FEP1/IMA3 and FEP3/IMA1 proteins inhibit the repression of bHLH factors by BTS. We had postulated that –Fe-regulated co-expression clusters provide new information about regulatory protein interaction complexes. Here, we report a targeted yeast two-hybrid screen among 23 proteins of the –Fe response. This identified a novel protein interactome involving another E3 ligase, namely BTSL1, basic helix-loop-helix (bHLH) protein POPEYE (PYE) and transcription factors of the subgroup IVc as well as FEP3/IMA1. Because of the difficulty in stable BTSL1 protein expression in plant cells, we used a yeast two hybrid-based deletion mapping, homology modeling and molecular docking, to pinpoint interaction sites in BTSL1 and FEP3/IMA1. bHLH IVc TFs have similar residues at their C-terminus as FEP3/IMA1 interacting sites. FEP3/IMA1 attenuated interaction of BTSL1 and bHLH proteins in a yeast three-hybrid assay, in line with physiological data pointing to enhanced Fe acquisition and allocation in FEP3/IMA1 overexpression and *btsl1 btsl2* mutant plants. Hence, exploiting –Fe-induced gene co-expression networks identified FEP3/IMA1 as a small effector protein that binds and inhibits the BTSL1 complex with PYE and bHLH subgroup IVc proteins. Structural analysis resolved interaction sites. This information helps improving models of Fe regulation and identifying novel targets for breeding of Fe-efficient crops.

## Graphical Abstract

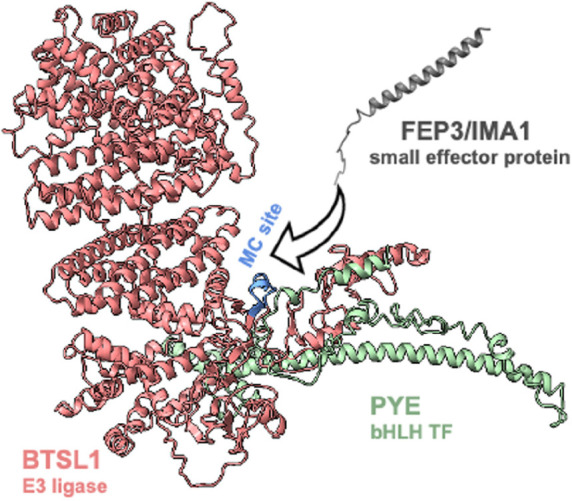

## Introduction

Generating resilient and biofortified crops for climate change and human nutrition requirements is one of the most challenging tasks for the future. The micronutrient iron (Fe) is a crucial cofactor for plant growth as it is needed for many redox and electron transfer reactions like those involved in chlorophyll synthesis and photosynthesis. Although very abundant in the soil, Fe is often not readily bio-accessible, because at neutral or basic pH it precipitates as insoluble Fe(III) oxides and hydroxides (Lindsay, 1988; Hans Wedepohl, 1995), and this will likely increase drastically with global warming and drought. To cope with low Fe bio-availability, plants mobilize Fe in the soil, either via Fe^3+^-chelating phytosiderophores (in grasses, so-called Strategy II) or via acidification and reduction of Fe^3+^ into Fe^2+^ (Strategy I) (Marschner and Römheld, 1994). Additionally, plants mobilize internal sources of Fe and transport it toward the shoots (Connorton et al., 2017). Fe deficiency stress results in leaf chlorosis and poor growth. Elevated cellular Fe, instead, may generate radicals via the Fe-catalyzed Fenton reaction, leading to unspecific damage of cellular components. Fe homeostasis is thus very critical for the performance of plants. Plants constantly adjust Fe mobilization with plant growth and environmental stress factors (Brumbarova et al., 2015). Clearly, plants sense the Fe status, signal their demand and regulate internal allocation and uptake of external Fe. The need to orchestrate these different processes is reflected in the complex transcriptomic network of genes that are co-regulated under Fe deficiency (–Fe) and that encode metal ion transporters, enzymes for reduction and chelation of Fe, transcription factors (TFs) and other regulators that steer the coordinated Fe deficiency response (Ivanov et al., 2012; Schwarz and Bauer, 2020). The co-expression clusters allow feed-forward and feed-back regulation of Fe mobilization, and there are many open questions as to the complex interconnections and regulatory mechanisms of the encoded proteins.

A cascade of basic helix-loop-helix (bHLH) transcription factors (TFs) that is conserved at least within eudicots, up-regulates Fe acquisition in response to a –Fe signal (Gao et al., 2019b). Arabidopsis (*Arabidopsis thaliana*) has at least 12 bHLH proteins controlling Fe uptake (Gao et al., 2019b; Schwarz and Bauer, 2020). bHLH proteins have been classified into subgroups according to their bHLH domain amino acid sequences (Heim et al., 2003). Subgroup IVc bHLH TFs (bHLH034, bHLH104, ILR3/bHLH105, bHLH115) have redundant functions. Together with bHLH subgroup IVb protein URI/bHLH121 they form heterodimeric complexes and induce the –Fe response (Zhang et al., 2015; Li et al., 2016; Liang et al., 2017; Gao et al., 2019a; Kim et al., 2019). bHLH011, another bHLH IVb TF, acts as a negative regulator of the Fe uptake machinery (Tanabe et al., 2019; Li et al., 2022). A third bHLH subgroup IVb protein named POPEYE (PYE) is also a negative regulator that down-regulates Fe distribution genes *NICOTIANAMINE SYNTHASE4* (*NAS4*), *FRO3* and *ZINC-INDUCED FACILITATOR1* (*ZIF1*) (Long et al., 2010). URI and bHLH IVc TFs up-regulate *PYE* and other co-expressed genes including *BHLH* Ib genes (Zhang et al., 2015; Liang et al., 2017; Gao et al., 2019a; Kim et al., 2019). –Fe-induced bHLH subgroup Ib proteins (bHLH038, bHLH039, bHLH100, bHLH101) form dimers with the bHLH protein FIT, and altogether they are essential for up-regulating Fe acquisition (Colangelo and Guerinot, 2004; Jakoby et al., 2004; Yuan et al., 2008; Wang et al., 2013; Trofimov et al., 2019; Cai et al., 2021). Root Fe acquisition involves the activation of *FERRIC REDUCTION OXIDASE2* (*FRO2*) encoding the Fe reductase that reduces Fe^3+^ to Fe^2+^ (Robinson et al., 1999) and *IRON-REGULATED TRANSPORTER1* (*IRT1*), which codes for the importer of Fe^2+^ (Vert et al., 2002).

The action of several bHLH subgroup IVb and IVc transcription factors is counteracted by E3 ligases that are induced by the bHLH cascade. ILR3 and bHLH115 are controlled through proteasomal degradation by their own target BRUTUS (BTS), a negative regulator of Fe uptake (Selote et al., 2015; Liang et al., 2017; Li et al., 2021). bHLH104 also interacts with BTS (Long et al., 2010; Selote et al., 2015). This paradoxical situation of incoherent regulation was explained by the need to have a shut-down mechanism for Fe re-mobilization (Hindt et al., 2017). Another hypothesis is that BTS ensures a constant turnover of TF (Selote et al., 2015). BTS has an interesting domain structure that indicates Fe-sensing functions. BTS has a C-terminal REALLY INTERESTING NEW GENE (RING) domain with E3 ligase activity and N-terminal hemerythrin/HHE-like cation-binding motifs for Fe and oxygen binding (Kobayashi et al., 2013; Selote et al., 2015). The two homologs BTS-LIKE1 (BTSL1) and BTSL2, which negatively regulate Fe uptake, have partly redundant functions, but only BTS is expressed in roots and shoots, while BTSL1 and BTSL2 are root-specific (Hindt et al., 2017). BTSL2 is tightly co-regulated with FIT, while BTSL1 is most similarly co-regulated with FIT target genes and Fe homeostasis genes for Fe allocation (Schwarz and Bauer, 2020). It was reported that FIT was degraded in the presence of BTSL2 (Rodriguez-Celma et al., 2019). What makes BTS/BTSL proteins so interesting is that they resemble FBXL5, a component of the mammalian Fe-sensing E3 ligase complex (Kobayashi et al., 2013). BTS protein stability and function are also coupled to Fe presence or absence. However, unlike FBXL5, BTS was found to be unstable in the presence of Fe (Selote et al., 2015).

A third level of regulation is exerted by small proteins. BTS was found to ubiquitinate a co-expressed and –Fe-regulated small Fe-uptake-promoting regulatory protein FE UPTAKE-INDUCING PEPTIDE3 (FEP3)/IRON MAN1 (IMA1) (Li et al., 2021). FEP/IMA are an interesting class of potential phloem-mobile small proteins. They share a 17-amino-acid C-terminal consensus sequence (Grillet et al., 2018; Hirayama et al., 2018). FEP/IMA small proteins induce Fe acquisition (Kobayashi et al., 2020), and indeed they are functionally interchangeable between species, showing that there must be likely a conserved mechanism of action (Grillet et al., 2018). When our work was initiated, no mechanism of action of FEP/IMA proteins had been known. In parallel to our work, just recently, it was described that FEP1/IMA3 and FEP3/IMA1 stabilized bHLH115 and ILR3 in the presence of BTS (Li et al., 2021). FEP/IMA proteins inhibited BTS-mediated degradation of the Fe deficiency response-inducing bHLH subgroup IVc proteins (Li et al., 2021). Interestingly, the bHLH factors have a C-terminal stretch resembling the FEP/IMA interaction region in BTS (Li et al., 2021). From this work, the question remained whether FEP/IMA proteins target BTSL proteins in similar manner. It was also unclear whether bHLH subgroup IVc TFs are targets of BTSL proteins. Moreover, little information has been available on protein complex structural aspects that explain the physiological data.

Transcriptional co-regulation in response to –Fe can be an indicator of protein-protein interaction, e.g., in the case of bHLH039/FIT (Yuan et al., 2008). We deciphered that –Fe-regulated co-expression gene clusters and their regulators serve to identify novel protein interaction complexes. We report here, that we tested protein interactions among 23 proteins of the –Fe response. Among them, we identified an interactome involving the proteins BTSL1, FEP3/IMA1, PYE, and bHLH subgroup IVc TFs. Analysis of this protein interactome showed that FEP3/IMA1 is an effector modulating the BTSL1-bHLH protein interaction.

## Results

### A Targeted Yeast Two-Hybrid Screen Uncovered the BTSL1-bHLH-FEP3/IMA1 Interactome

To identify novel protein interaction complexes, we exploited co-expression network information from Fe deficiency transcriptomics data sets (Ivanov et al., 2012; Schwarz and Bauer, 2020) and literature to select 23 candidates for a targeted yeast two-hybrid (Y2H)-based pairwise protein interaction screen, hereafter termed targeted Y2H screen (Supplementary Table 1). Criteria for the selection of candidates were (i) unknown functions of cytosolic proteins during Fe deficiency responses (at the time the study was initiated) and (ii) known regulatory functions of Fe homeostasis in the cytosol or nucleus, including proteins from outside of this network and enzymatic functions. Because of the redundancy of bHLH subgroup Ib proteins, we had limited this group to bHLH039, which has the strongest effect among the four proteins (Wang et al., 2013; Trofimov et al., 2019).

**FIGURE 1:**
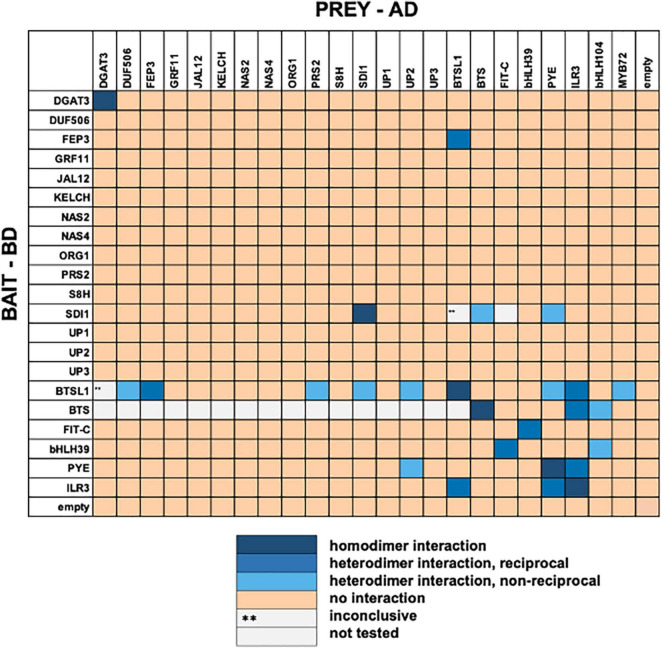
Summary results of targeted yeast two hybrid (Y2H) screen. Twenty three protein candidates (Supplementary Table 1) were tested reciprocally in pairwise combinations in a targeted Y2H screen. Bait protein was fused to the GAL4 DNA-binding domain (BD), prey protein to the GAL4 activation domain (AD). The color code distinguishes homodimeric interaction (dark blue), reciprocal heterodimeric interaction (middle blue), non-reciprocal heterodimeric interaction (light blue), no interaction (light orange) or inconclusive and non-tested interactions (^**^, light gray). bHLH104 and Myb72 were not included as bait because of autoactivation. FIT-C was included as it is not auto-activating (Gratz et al., 2020). Original data are presented in Supplementary Figurees 1, 2.

At first, all 23 candidates were tested in pairwise combinations in the targeted Y2H screen (Supplementary Table 1 and Supplementary Figurees 1, 2). If possible, we performed reciprocal (AD/BD and vice versa) combinations and included homodimeric interaction tests. 5–6% of tested interactions were positive and they comprised 20 heterodimeric and six homodimeric interactions (summarized in Figure 1). We noted that several interactions involved BTS or BTSL proteins and bHLH factors. For example, BTSL1 interacted with PYE, ILR3, MYB72, DUF506, SDI1, PRS2, FEP3/IMA1, and UP2. Besides BTSL1, PYE interacted with UP2 and SDI1. Hence, the targeted Y2H screen provided evidence for a Fe-regulatory interactome involving PYE and bHLH subgroup IVc TFs together with FEP3/IMA1 and BTSL1. Additionally, the connection between Fe, sulfur and glucosinate metabolism via SDI interactions is suggested from the screen (Aarabi et al., 2016; Samira et al., 2018). Among the interacting pairs, we detected the expected interactions FIT + bHLH039 (Yuan et al., 2008), BTS + ILR3 (Long et al., 2010), BTS + bHLH104 (Long et al., 2010), PYE + ILR3 (Selote et al., 2015), and ILR3 + ILR3 (Li et al., 2016). The latter was found negative in another study (Selote et al., 2015). Some reported protein interactions were not picked up in our screen, bHLH104 + ILR3 (Zhang et al., 2015), FIT + BTSL2 (Rodriguez-Celma et al., 2019). On the other hand, we picked up an interaction that had been found to be negative in one previous study, namely PYE + PYE (Selote et al., 2015). The cases of interacting or non-interacting pairs, that were different from the literature can be explained by different protein fusion constructs used and technical aspects related to different experimental Y2H procedures. For example, FEP3/IMA1 interaction with BTS was previously detected using adenine selection in the Y2H assay (Li et al., 2021). Instead, our study used histidine selection and supplementation with 3AT to show that FEP3/IMA1 interacted with BTSL1 and BTSL2 but not BTS. Presumably, the stringency of Y2H conditions impacted colony growth and protein interaction.

In summary, the screen with 23 protein candidates uncovered 19 previously not known heterodimer and five homodimer interactions. We decided to focus on the BTSL1-bHLH-FEP3/IMA1 interactome. At the time the screen was conducted a mechanism of action of BTSL and FEP3/IMA1 had not been known, and we postulated a regulatory protein interaction.

### Evidence for the BTSL1-bHLH-FEP3/IMA1 Interactome Was Further Studied in Targeted Assays

**FIGURE 2:**
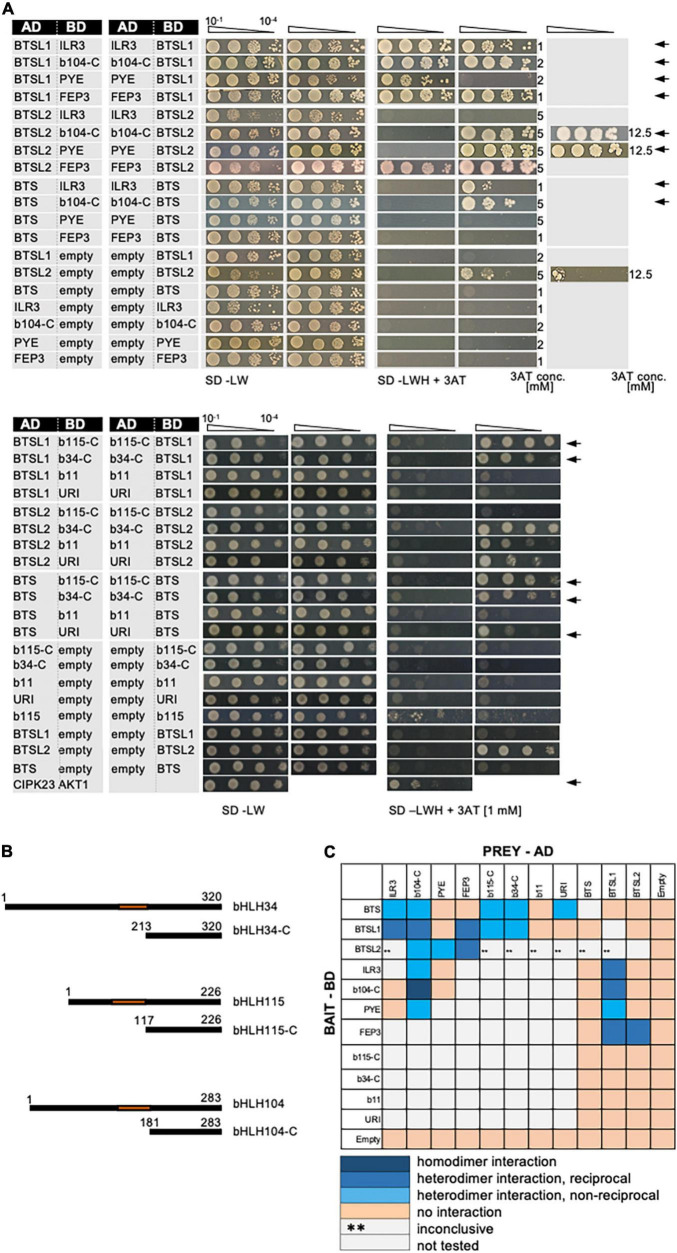
Validation of the BTS/L-bHLH-FEP3/IMA1 interactome. **(A)** BTSL1 and BTSL2 were tested in reciprocal targeted Y2H assays against various bHLH proteins of the subgroups IVb and IVc and FEP3. Yeast co-transformed with the AD and BD combinations were spotted in 10-fold dilution series (A_600_ = 10^–1^–10^–4^) on SD-LW (transformation control) and SD-LWH plates supplemented with different concentrations (conc.) of 3AT as indicated on the right side (selection for protein interaction). Negative controls: empty vectors. Positive control: CIPK23 and AKT1. Arrows indicate interaction. **(B)** Schematic representation of full-length bHLH34, bHLH115, bHLH104 and their respective C-terminal parts used for Y2H. C-terminal parts lack the N-terminus and the DNA-binding domains (represented in orange). **(C)** Summary results of A. The color code distinguishes homodimeric interaction (dark blue), reciprocal heterodimeric interaction (middle blue), non-reciprocal heterodimeric interaction (light blue), no interaction (light orange) or inconclusive and non-tested interactions (^**^, light gray). Additional controls and Y2H validation data are presented in Supplementary Figuree 3.

The discovered BTSL1-bHLH-FEP3/IMA1 interactome attracted our attention as it suggested mechanistic insight into the functions of these proteins in the context of bHLH TF action. In the validation experiments we included all TFs of the bHLH subgroup IVb and IVc group as their roles in Fe regulation were being revealed (Figure 2 and Supplementary Figuree 3). Deletion constructs were included to avoid auto-activation in the case of bHLH104-C, bHLH115-C and bHLH034-C (Figures 2A,B). BTS, BTSL1 and BTSL2 were tested against all bHLH proteins of the subgroups IVb and IVc (Figures 2A,B) and each other (Supplementary Figuree 3A). bHLH proteins ILR3, bHLH104-C and PYE were also tested against each other (Supplementary Figuree 3B). This way, we found the following positive interactions: BTS interacted with ILR3, bHLH104-C, bHLH115-C, bHLH034-C and URI (Figure 2), confirming the interactions of BTS with bHLH104, ILR3 and bHLH115 (Long et al., 2010; Selote et al., 2015; Li et al., 2021). Previously no interaction was found for BTS-URI (Long et al., 2010; Gao et al., 2019a) and BTS-bHLH034-C (Long et al., 2010). We did not find evidence for interaction of BTS with PYE, FEP3/IMA1, bHLH011, BTSL1, or BTSL2 (Figure 2), confirming published data that BTS does not interact with PYE (Long et al., 2010; Selote et al., 2015) and bHLH011 (Long et al., 2010). As mentioned above, BTS had been found to interact with FEP3/IMA1 by Li et al. (2021). BTSL1 interacted in our study with ILR3, bHLH104-C, PYE, FEP3/IMA1, bHLH115-C, and bHLH034-C (Figure 2). BTSL1 did not interact with bHLH011, URI, BTS and BTSL2. BTSL2 interacted with bHLH104-C, PYE and FEP3/IMA1 (Figure 2), while it did not interact with ILR3, BTS, and BTSL1. In a previous study, URI was also not found to interact with BTSL1 or BTSL2 (Gao et al., 2019a). We found that bHLH104-C interacted additionally with ILR3, PYE and these factors homodimerized (Supplementary Figuree 3B). Hence, bHLH104-C indeed interacts with ILR3 (Li et al., 2016). bHLH104 homodimerizes as previously found (Li et al., 2016), and bHLH104 interacts with PYE (Selote et al., 2015). Since it was reported that BTSL1 and BTSL2 interact with FIT, but Y2H data were not yet provided (Rodriguez-Celma et al., 2019), we also re-tested specifically the interaction of BTSL1 and BTSL2 with full-length FIT and bHLH039. However, as in the targeted Y2H screen above, we did not find any proof in any combination for BTSL1 or BTSL2 interaction with FIT nor bHLH039, even though both proteins worked successfully as FIT/bHLH039 interaction pair (Supplementary Figurees 3C,D).

Taken together, the BTSL1-bHLH-FEP3/IMA1 interactome was confirmed by targeted and extended Y2H data (summarized in Figure 2C). Some proteins within this group had a large set of interaction partners, e.g., bHLH104-C and BTSL1. Others had only one or none, e.g., URI and bHLH011. This shows specificity at the level of protein interactions. The interaction of BTSL1 with FEP3/IMA1 had been particularly exciting since this had offered the possibility of uncovering a novel mechanism of action for FEP3/IMA1 and BTSL1. We also focused on interacting bHLH proteins ILR3, bHLH104 and PYE. The C-termini of some of the transcription factors were sufficient for protein interactions. This was not surprising since the C-termini were also found sufficient for interactions of FIT-C (Gratz et al., 2020), bHLH034-C, bHLH104-C and ILR3-C (Li et al., 2016). Thus, BTSL1-bHLH interactions did not rely on the canonical bHLH domain.

Full-length BTS protein is unstable (Selote et al., 2015), and we suspected that because of the similar structures this might also be the case for BTSL1 and BTSL2. A reliable assay of bimolecular fluorescence complementation (BiFC) of YFP consists in simultaneous mRFP expression as control of transformation to validate novel protein interactions of Fe-regulated proteins in plant cells (Gratz et al., 2019; Khan et al., 2019). When we applied this method to study BTS/BTSL protein interactions, we detected the interaction between nY-BTSL1 and cY-PYE in a few of the transformed cells (Supplementary Figuree 4A, top). It was not possible to detect interactions between other fusion proteins of BTSL1 and ILR3, bHLH104 or FEP3/IMA1, or any of the BTSL2 fusion proteins. In these negative cases, mRFP was detected as a control, indicating that transformation had worked (data not shown). The C-terminal part of BTS-C contains CHY- and CTCHY-type zinc (Zn) finger domains, a Zn ribbon domain and RING with E3 ligase function, and it was found sufficient for tested bHLH interactions (Selote et al., 2015). After switching to a comparable form of BTSL1-C we detected again an interaction with PYE by BiFC in a few cells (Supplementary Figuree 4A, middle, nY-BTSL1-C + cY-PYE). mRFP signals were always present in all cells of the transformed region of the leaves (Supplementary Figuree 4A). Interestingly, the YFP signals were present at the cell periphery rather than in the nucleus for full-length BTSL1 + PYE, while YFP signals were present in the nucleus for BTSL1-C + PYE fluorescence protein fusion (Supplementary Figuree 4A, compare top and middle). Additionally, the interactions of BTSL1-C + ILR3 were detected in the nucleus (Supplementary Figuree 4A bottom, nY-ILR3/cY-BTSL1-C; Supplementary Figuree 4B). No other protein interactions could be confirmed by this method, also not FIT-BTSL1C, while mRFP was visible as positive transformation control in all cases (Supplementary Figuree 4C, nYFP-FIT together with cY-BTSL1-C and -BTSL2-C). Overall, negative BTSL1 and BTSL2 data have to be carefully interpreted because of the low success rate for detecting protein interaction of BTSL1 and BTSL2 via BiFC, which was presumably due to their low stability.

In summary, the novel protein-protein interactions of the BTSL1-bHLH-FEP3/IMA1 interactome were also found by targeted Y2H assays. Plant cell BiFC, although a reliable assay with positive and negative controls, was not suited to confirm all protein interactions since YFP signals representing BTSL1 interactions were detected in only a few cells of the transformed regions. The differential localization of protein complexes BTSL1 + PYE and BTSL1-C + PYE by BiFC inside the cells showed, however, that the obtained few YFP signals were not artifacts. Presumably, it was difficult to study BTSL1 protein interactions by BiFC in plant cells because of plant factors that render the proteins unstable, as reported previously for BTS (Selote et al., 2018).

Previous PYE, ILR3, and FEP3/IMA1 fluorescent fusion protein studies showed that these proteins can be present in the same root cells in response to –Fe (Long et al., 2010; Grillet et al., 2018; Samira et al., 2018; Tissot et al., 2019). We specifically intended to localize and co-localize BTSL proteins and their interaction partners in plant cells. However, as for BiFC, these studies were also hampered by the low detection of BTSL fluorescent fusion proteins. We were only able to study intracellular localization qualitatively. Surprisingly, fluorescence protein-tagged BTSL1 localized mainly at the cell periphery and only weakly to the nucleus, as observed before in BiFC (Supplementary Figuree 4D, YFP-BTSL1, compare with Supplementary Figuree 4A). The fluorophore position (N-/C-terminal) did not affect BTSL1 localization (Supplementary Figuree 5). YFP-tagged BTSL2 localized to the nucleus and the cytoplasm (Supplementary Figuree 4D, YFP-BTSL2). Remarkably, BTSL1-C-GFP and BTSL2-C-GFP localized more to the nucleus and less to the cytoplasm compared to the full-length version, again matching the BiFC data (Supplementary Figuree 4D, compare BTSL1-C-GFP, BTSL2-C-GFP with YFP-BTSL1 and -BTSL2, and compare with Supplementary Figuree 4A), indicating an interesting pattern of BTSL1 and BTSL2 localization with an unexpected role of the N-terminal HHE domains in steering the intracellular localization. In contrast, YFP-BTS was localized exclusively to the nucleus (Supplementary Figuree 4D, YFP-BTS). FEP3-GFP was localized to the nucleus and cytoplasm, with a preference for the cytoplasm (Supplementary Figuree 4, FEP3-GFP). PYE-GFP, ILR3-GFP and YFP-bHLH104 were located in the nucleus as expected for the transcription factors (Supplementary Figuree 4D, PYE-GFP, ILR3-GFP, YFP-bHLH104), also in accordance with previous reports (Long et al., 2010; Li et al., 2016; Samira et al., 2018). BTSL1-mCherry co-localized with ILR3-GFP in the nucleus and the same was found for BTSL1-GFP and PYE-mCherry as well as YFP-BTSL2 and PYE-mCherry (Supplementary Figuree 4E). BTSL1-GFP and PYE-mCherry also co-localized outside the nucleus, whereby the PYE-mCherry signal at the cell periphery was weak compared with the nuclear signal (Supplementary Figuree 4E, compare cell1 and cell 2). It was not possible to obtain fluorescence signals for BTSL1-mCherry when it was co-expressed with FEP3-GFP (not shown). Again, as was the case for BiFC, protein fluorescence detection of BTSL1 and BTSL2 was hampered by low detection of signals. In summary, these results indicate that proteins of the BTSL1-bHLH-FEP3/IMA1 interactome localize to large extent in plant cells. Aside from the nucleus interesting dynamic cytoplasmic and cell peripheral localization effects were noted for BTSL1 and BTSL2, dependent on the N-terminal part with HHE domains.

We also verified that the genes encoding the BTSL1-bHLH-FEP3/IMA1 interactome were co-expressed in the similar root cells when respective transgenic GUS plants were grown in parallel. All tested promoter-reporter activities were present in the root differentiation zone where Fe uptake occurs (Supplementary Figuree 6). *BTSL1* promoter was mainly active in the outer root layers, in contrast to its proposed interaction partners encoded by *ILR3*, *BHLH104*, and *FEP3*, which were expressed predominantly in the root stele. Interestingly, the *BTSL1* expression pattern overlapped with the *PYE* expression pattern in the root differentiation zone in our analysis (Supplementary Figuree 6). Hence, the tissue-specific GUS staining patterns we detected for all promoters in our growth conditions confirmed previous reports about the root-zone-related promoter regulation (Long et al., 2010; Li et al., 2016; Liang et al., 2017; Grillet et al., 2018; Samira et al., 2018; Rodriguez-Celma et al., 2019). The promoter activity does not necessarily restrict the protein to the same location. For example, FEP3/IMA1 moved long-distance from shoot to root in grafting experiments, and PYE-GFP and ILR3-GFP were located in all root tissues across the root when expressed from their promoters while the same promoter fragments conferred reporter activity mainly in the stele of the mature root zone (Long et al., 2010; Grillet et al., 2018; Samira et al., 2018).

Taken together, protein complexes of the BTSL1-bHLH-FEP3/IMA1 interactome can be formed in root cells.

### Interaction Sites Between BTSL1, bHLH Proteins, and FEP3/IMA1 Were Mapped

The BTSL1-bHLH-FEP3/IMA1 interactome was very exciting and it was then interesting to better resolve the interaction. Because of the limited success in BTSL1 protein expression in plant cells, Y2H was the method of choice. The synthetic model yeast cell system is a heterologous system, broadly utilized to fine-map interaction sites in the absence of interfering plant factors in cells.

**FIGURE 3:**
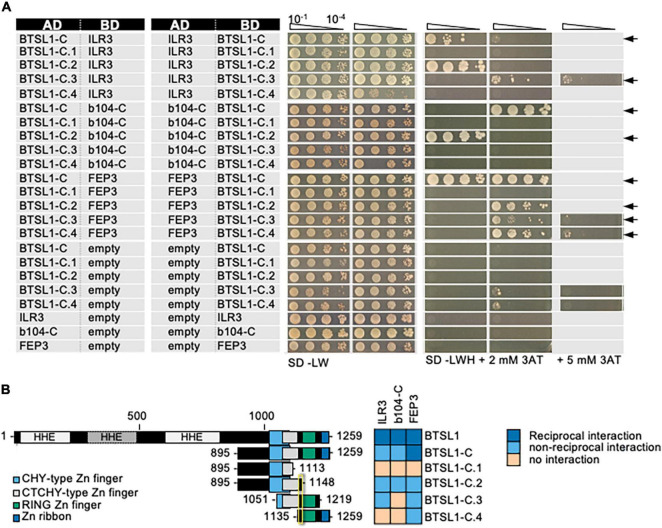
Mapping of the interaction sites in BTSL1 by yeast two hybrid (Y2H) assays. BTSL1-C and deletion forms of BTSL1-C were tested in reciprocal targeted Y2H assays against ILR3, bHLH104-C (b104-C) and FEP3/IMA1. Yeast co-transformed with the AD and BD combinations were spotted in 10-fold dilution series (A_600_ = 10^–1^–10^–4^) on SD-LW (transformation control) and SD-LWH plates supplemented with different concentrations of 3AT as indicated (selection for protein interaction). Negative controls: empty vectors. Arrows indicate interaction. **(B)** Schematic representation and summary of Y2H results. Left, schematic representation of full-length BTSL1, BTSL1-C and deletion constructs of BTSL1-C.1 to –C.4, used for Y2H. The domains are indicated in color. The yellow box highlights the mapped interaction site for interaction with bHLH proteins and FEP3/IMA1. Right, summary results of panel **(A)**. The color code distinguishes reciprocal positive interactions (dark blue), non-reciprocal positive interactions (light blue), negative results on interactions (light orange).

First, we mapped the required interaction site of BTSL1. It was suggested that BTS interacts with its target TFs ILR3 and bHLH115 via the RING domain (Selote et al., 2018). BTSL1 and likewise BTSL1-C interacted with bHLH factors and FEP3/IMA1 suggesting that the RING domain may also be important for BTSL1 (Figure 3, see also Figure 2). To pinpoint the specific interaction site, we used a deletion mutant approach and delimited further the C-terminal region of BTSL1 that is required for interaction with ILR3, bHLH104-C and FEP3/IMA1 (Figure 3). We divided BTSL1-C into further four deletion forms (BTSL1-C1 to -C.4, Figure 3B). BTSL1-C.1, lacking RING, Zn ribbon and the full CTCHY region, was not able to interact with ILR3, bHLH104-C or FEP3/IMA1 (Figure 3). The slightly longer form BTSL1-C.2 with CHY and CTCHY domains lacked RING and Zn ribbon domains and interacted with ILR3, bHLH104-C and FEP3/IMA1 (Figure 3). This indicates that BTSL1 RING and Zn ribbon are not needed for this interaction. A further deletion construct BTSL1-C.3 contained only the CTCHY plus RING domains, and it interacted with ILR3 and FEP3/IMA1, but not with bHLH104-C (Figure 3). This shows that the full CTCHY plus RING are sufficient for interaction with FEP3/IMA1 and ILR3, but not for interaction with bHLH104-C. Instead, the construct BTSL1-C.4 with only RING and Zn ribbon interacted with FEP3/IMA1 but none of the TF proteins (Figure 3). In summary, the three deletion constructs that suggest protein interaction with FEP3/IMA1 (BTSL1-C.2 to -C.4) had one common small region of 14 amino acids (aa). This small 14 aa-region was named M-C site according to the first and last aa of this 14-aa stretch. M-C is located between CTCHY and RING (Figure 3B, yellow box). Results were less clear for binding of ILR3 and bHLH104 to BTSL1, but CTCHY and CHY domains were needed for the interaction.

**FIGURE 4:**
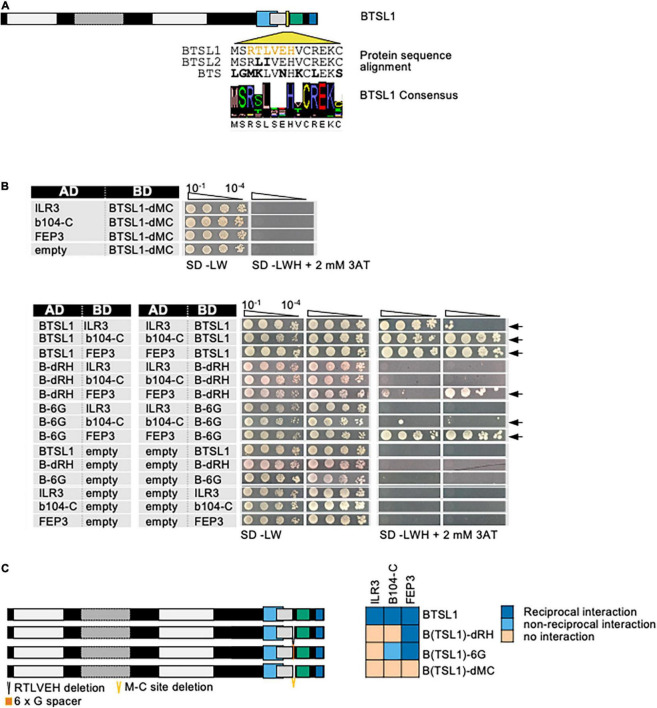
Fine-mapping of the interaction sites in BTSL1 by yeast two hybrid (Y2H) assays. **(A)** Protein structure of BTSL1 and protein sequence alignment of the interaction M-C site within the yellow-boxed region that is highlighted, see also Figure 3B. A further sub-region is marked in orange letters, R-H site. The consensus sequence was obtained by identifying orthologs using blastp and comparing the sequences of the 100 best hits. **(B)** Small targeted deletion forms of BTSL1-C of or within the M-C site were tested in reciprocal targeted Y2H assays against ILR3, bHLH104-C (b104-C) and FEP3/IMA1. Yeast co-transformed with the AD and BD combinations were spotted in 10-fold dilution series (A_600_ = 10^–1^–10^–4^) on SD-LW (transformation control) and SD-LWH plates supplemented with 3AT as indicated (selection for protein interaction). Negative controls: empty vectors. Arrows indicate interaction. **(C)** Schematic representation and summary of Y2H results. Left, schematic representation of full-length BTSL1 and deletion constructs of and within the M-C site, used for Y2H. B-6G is a sextuple glycine spacer. The regions are indicated in color. Right, summary results of panel **(B)**. The color code distinguishes reciprocal positive interactions (dark blue), non-reciprocal positive interactions (light blue), negative results on interactions (light orange).

The BTSL1 M-C site was investigated in more detail and a consensus sequence within related Viridiplantae orthologs was identified (Figure 4A, indicated by yellow arrowhead). Deleting the M-C site in BTSL1-C (BTSL1C-dMC) abolished interactions with TFs ILR3 and bHLH104-C and FEP3/IMA1, indicating that the M-C site was essential (Figures 4B,C). An internal R-H part, named according to the first and last aa of an internal part, was more variable (Figures 4A,C, indicated in yellow), and we found that by deleting it (BTSL1-dRH), the interaction was still possible with FEP3/IMA1, but not with TFs (Figures 4B,C). Substituting as control the R-H part with a sextuple G residue spacer (BTSL1-6G) also resulted in interaction with FEP3/IMA1 but did not restore interaction with ILR3 and bHLH104-C (Figures 4B,C). Possibly, the evolutionarily conserved aa adjacent to R-H is important for interaction with FEP3/IMA1 (Figure 4A). Thus, FEP3/IMA1 interacts with BTSL1 at a position that is close to the interaction site of ILR3 and bHLH104-C. In summary, the 14-aa M-C site located close to the BTSL1 E3 RING domain is needed for interaction with FEP3, ILR3 and bHLH104-C. Within this region, the R-H part is essential for interaction with ILR3 and bHLH104-C, but not with FEP3/IMA1. This indicates that FEP3/IMA1 and the TFs do not bind identically to BTSL1.

**FIGURE 5:**
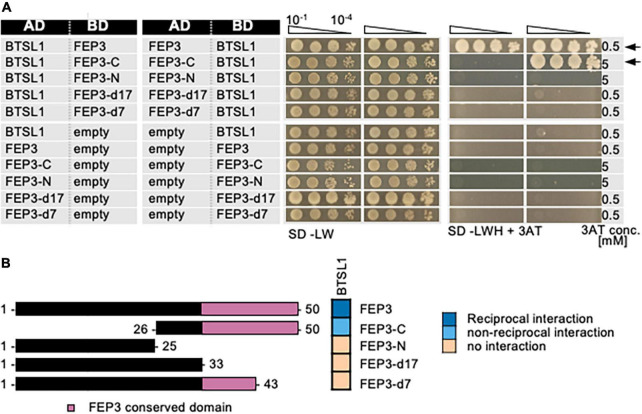
Mapping of the interaction site in FEP3/IMA1 by yeast two hybrid (Y2H) assays. **(A)** Targeted deletion forms of FEP3/IMA1 were tested in reciprocal targeted Y2H assays against BTSL1. Yeast co-transformed with the AD and BD combinations were spotted in 10-fold dilution series (A_600_ = 10^–1^–10^–4^) on SD-LW (transformation control) and SD-LWH plates supplemented with different 3AT concentrations (conc.) as indicated (selection for protein interaction). Negative controls: empty vectors. Arrows indicate interaction. **(B)** Schematic representation and summary of Y2H results. Left, schematic representation of FEP3 and deletion constructs, used for Y2H. Pink color illustrates the conserved region of FEP3 (see Supplementary Figuree 5). Right, summary results of panel **(A)**. The color code distinguishes reciprocal positive interactions (dark blue), non-reciprocal positive interactions (light blue), negative results on interactions (light orange).

Second, the interaction site within FEP3/IMA1 was mapped. As shown in previous data on FEP sequence conservation across the plant kingdom (Grillet et al., 2018), FEP3/IMA1 protein has the conserved stretch of final 17 aa at its C-terminus (Supplementary Figuree 7). The N-terminal half and a C-terminal half of FEP3/IMA1 (termed FEP3-N and FEP3-C) were tested for their ability to interact with BTSL1, and only FEP3-C was found to be the interacting part (Figure 5). Next, two truncated FEP3/IMA1 versions lacking the conserved stretch (FEP3-d17) or lacking the last seven aa YDYAPAA (FEP3-d7) were tested (Figure 5). Neither of the two constructs interacted with BTSL1, showing that the conserved stretch and the last seven aa in FEP3/IMA1 are crucial for FEP3/IMA1 interactions.

**FIGURE 6:**
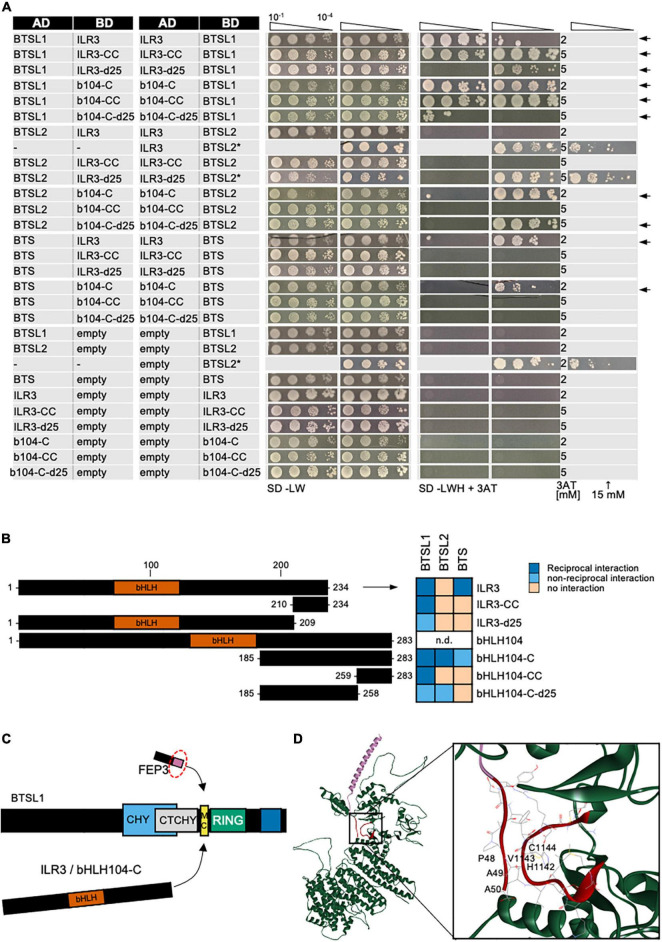
Mapping of the interaction site in bHLH subgroup IVc proteins ILR3 and bHLH104 by yeast two hybrid (Y2H) assays. **(A)** Targeted deletion forms of bHLH proteins were tested in reciprocal targeted Y2H assays against BTSL1. Yeast co-transformed with the AD and BD combinations were spotted in 10-fold dilution series (A_600_ = 10^–1^–10^–4^) on SD-LW (transformation control) and SD-LWH plates supplemented with different 3AT concentrations (conc.) as indicated (selection for protein interaction). Negative controls: empty vectors. Arrows indicate interaction. BTSL2* and BTSL2 refer to two separate controls. **(B)** Schematic representation and summary of Y2H results. Left, schematic representation of bHLH and deletion constructs, used for Y2H. The color illustrates the proposed region of similarity with the C-terminus of FEP3/IMA1 (see Supplementary Figuree 8). Right, summary results of panel **(A)**. The color code distinguishes reciprocal positive interactions (dark blue), non-reciprocal positive interactions (light blue), negative results on interactions (light orange). **(C)** Proposed mechanistic model of BTSL1-C interaction at the fine-mapped M-C site with bHLH proteins of subgroup IVb and IVc and the C-terminal conserved region of FEP3/IMA1. Compare with Figures 4–6 for depicted functional domains. **(D)**, Molecular homology modeling and molecular docking of BTSL1 and FEP3/IMA1. Left, Homology model of BTSL1 protein predicted using AlphaFold2, used for molecular docking with FEP3. Right, details of molecular docking model between BTSL1 and FEP3. The aa highlighted are HVC within the M-C region of BTSL1 and the PAA region at the C-terminus of FEP3 that shows similarity with the C-terminus of bHLH IVb and IVc proteins. The model suggests that FEP3 is an allosteric inhibitor of bHLH binding to BTSL1.

Third, we mapped the interaction site within the C-terminal regions of bHLH IVc proteins ILR3 and bHLH104 with BTSL1 and compared them with BTSL2 and BTS (Figure 6). As shown above, ILR3 interacted with BTSL1 and BTS, but not BTSL2, while bHLH104-C interacted with all three BTS/L proteins. We made an interesting observation by aligning the FEP3/IMA1 sequence with the C-termini of bHLH IVc protein sequences (Supplementary Figuree 8). We found rough similarities and conserved PAA/PVA motifs at the C-terminal ends of FEP3/IMA1 and the bHLH IVc TFs (Supplementary Figuree 8). In comparison, bHLH Ib protein C-termini did not align with FEP3/IMA1 (data not shown). We figured that one explanation for the protein interactions could be that FEP3/IMA1 mimics bHLH IVc proteins within their last 25 aa during interaction with BTSL1. To test this, we constructed ILR3-d25 and bHLH104-C-d25 that lacked the 25 aa-region aligning with FEP3/IMA1 YDYAPAA and tested their ability to interact with BTS/L proteins (Figures 6A,B). We found that ILR3-d25 fragment still interacted with BTSL1, but no longer with BTS, while bHLH104-C-d25 still interacted with BTSL1 and BTSL2 but also no longer with BTS. Interestingly, short fragments only consisting of the last 25 aa, ILR3-CC and bHLH104-CC, even tended to interact better with BTSL1 than the d25 fragments, while no interaction was found with BTSL2 or BTS (Figures 6A,B). Therefore, the last 25 aa of C-terminal ends of ILR3 and bHLH104 did not appear essential for interaction in all cases, but they were important. The importance of the last 25 aa was also reported in a study for the bHLH105 and bHLH115-BTS interaction (Li et al., 2021).

Taken together, we were able to map interaction sites for the BTSL1-bHLH-FEP3/IMA1 interactome (summarized in Figure 6C).

Homology modeling and molecular docking are today powerful tools that predict with high confidence protein and protein complex structures. Via AlphaFold, we obtained a protein structure that we used for theoretical molecular docking experiments considering free energy values between the mapped interaction sites of BTSL1 and FEP3. Interestingly, this theoretical approach underlined experimental data and indicated precisely the three aa residues HVC within the M-C site covering with H the last aa of the R-H site of BTSL1 (Figure 6D). The top model that emerged indicated that PAA of the last seven aa YDYAPAA of FEP3/IMA1 bind to BTSL1-HVC (Figure 6D). As described above, PAA/PVA residues are also contained at the C-terminal end of bHLH IVc factors (Supplementary Figuree 8). Thus, theoretical modeling fit with experimental evidence. Hence, through Y2H studies and molecular docking, the interaction sites relevant for the BTSL1-bHLH-FEP3/IMA1 interactome were fine-mapped.

### FEP3/IMA1 Attenuated the Interaction of BTSL1 With PYE and bHLH IVC Transcription Factors, Providing Evidence for an Effector Interaction

FEP3/IMA1 is a positive regulator of Fe uptake and potential phloem-mobile signal (Grillet et al., 2018). However, the mechanism by which FEP3/IMA1 acts had not been known. BTSL1 and BTSL2 are suspected Fe sensors and negative regulators of Fe uptake (Hindt et al., 2017). This hypothesis was strengthened by the observation, that two transgenic Arabidopsis lines over-expressing *FEP3/IMA1* (FEP3-OX#1 and #3) had similar physiological phenotypes as loss-of-function defects in *btsl1 btsl2* mutants when comparing them side-by-side in the same growth system (for characterization of lines see Supplementary Figuree 9). FEP3-Ox plants and *btsl1 btsl2* mutants had increased Fe contents per dry weight in seeds (Supplementary Figuree 10A), longer roots than wild type at –Fe and partly also at +Fe (Supplementary Figuree 10B). When examining the downstream responses of bHLH IVb and IVc TFs, *FEP3/IMA1* and *BTSL* gene expression followed the expected pattern in overexpression and mutant situations. Interestingly, *FEP3/IMA1* was not up-regulated in response to –Fe in *btsl1 btsl2* compared to wild type (Supplementary Figuree 11 upper row). In contrast, *BTS*, *BHLH038*, *BHLH039*, *PYE*, *FRO3*, and *NAS4* did not show the *FEP3/IMA1* expression pattern and hence they were not co-expressed with *FEP3/IMA1* in *btsl1 btsl2*. Instead, with exception of non-regulated *NAS4*, all these genes showed a tendency to be up-regulated in FEP3-Ox and *btsl1 btsl2* conditions compared to wild type, which was significant in the case of *BHLH038* in FEP3-Ox#1 at +Fe and in *btsl1 btsl2* at –Fe, for *BHLH039* in FEP3-Ox#3 at –Fe and in *btsl1 btsl2* at + and –Fe, for *PYE* in FEP3-Ox#1 at +Fe, and in none of the cases for *FRO3* (Supplementary Figuree 11 two middle rows). At the level of root Fe acquisition genes, *FIT* was not found differentially regulated in the mutant lines. *IRT1* and *FRO2* were co-expressed but significantly up-regulated only in the case of *IRT1* in *btsl1 btsl2* at –Fe (Supplementary Figuree 11 bottom row). Hence, except for *FEP3/IMA1* there was no other case of down-regulation. The gene expression patterns indicate that *FEP3*, *BTSL1* and *BTSL2* affect –Fe response regulation upstream of *BHLH* subgroup Ib genes at the level of bHLH IVc and URI regulation. *URI*, *BHLH011* and *BHLH115* expression did not differ between wild type and mutant lines. However, *ILR3*, *BHLH104*, and *BHLH034* were in some cases down-regulated in mutants, which was significant in the case of *ILR3* and *BHLH104* in FEP3-Ox#1 at + and –Fe, as well as in FEP3-Ox#3 at +Fe and in *btsl1 btsl2* at –Fe, and for *BHLH034* in FEP3-Ox#1 at +Fe (Supplementary Figuree 12). Thus, *ILR3*, *BHLH104*, and *BHLH034* were transcriptionally regulated in an opposite manner as their downstream targets *BHLH038*, *BHLH039*, and *PYE* in FEP3-OX lines.

Together, these data indicate that FEP3/IMA1 acts as a positive regulator in Fe uptake, supporting published data (Grillet et al., 2018), while BTSL1/BTSL2 are negative regulators. From the gene expression, these effects happen upstream of *BHLH039* and *PYE*, and *FEP3/IMA1* does not need to be highly expressed for Fe accumulation in *btsl1 btsl2*. One possibility is that the bHLH factors ILR3, bHLH104 and bHLH034, which are positive regulators of *BHLH* subgroup Ib genes, are themselves negatively regulated downstream of FEP3/IMA1, while FEP3/IMA1 is positively affected by BTSL1 and BTSL2. Further, it is possible that FEP3/IMA1 binds BTSL1 and thereby modulates the interaction of BTSL1 with bHLH proteins. These bHLH proteins may regulate each other. Such a model predicts that FEP3/IMA1 is an effector protein that prevents or reduces BTSL1-bHLH interaction.

**FIGURE 7:**
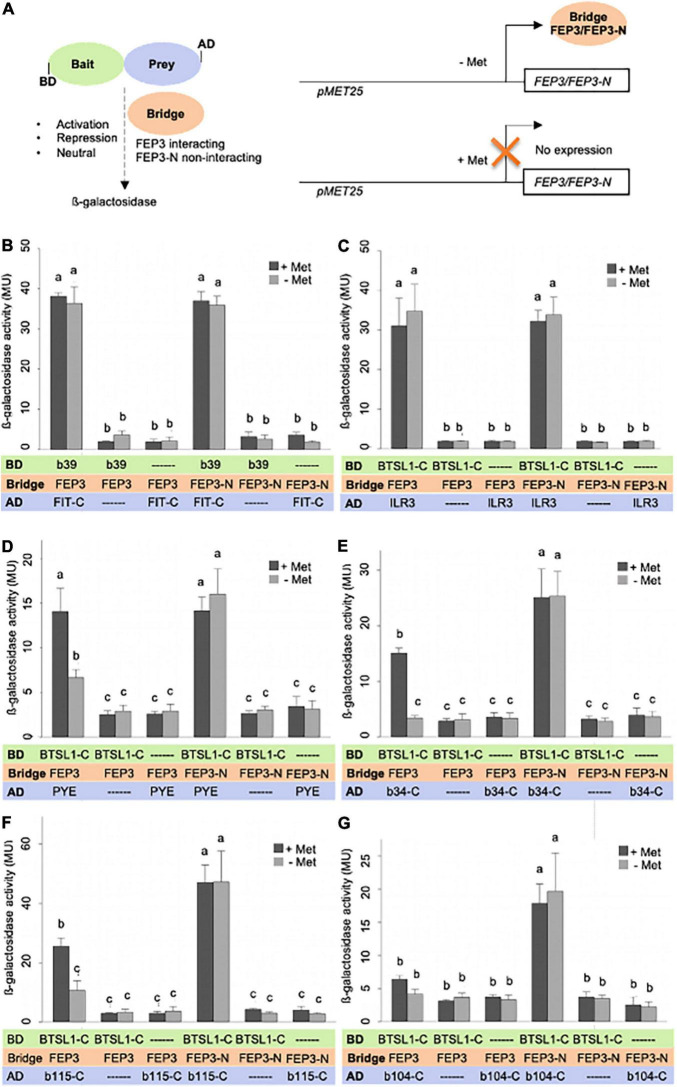
FEP3/IMA1 effect on BTSL1-C and bHLH IVb and IVc interaction quantified by yeast three hybrid (Y3H) assay. **(A)** Schematic representation of Y3H principle and design. Left, the protein interaction strength between a bait protein (fused with Gal4 DNA binding domain, BD) and prey protein (fused with Gal4 activation domain, AD) is measured by ß-galactosidase activity, here BTSL1-C and a bHLH protein (part). The effect of a bridge protein on protein interaction is measured, here interacting FEP3/IMA1 and negative control non-interacting FEP-N, leading to either activation, repression or neutral effect on bait-prey protein interactions. Note that the term “bridge” is a neutral term, and depending in the result the “bridge” protein may act as positive or negative effector protein or have a neutral effect. Right, the Bridge protein is expressed under a pMET25 promoter by supplementation with or without methionine (+Met, -et). **(B–G)**, Quantification of protein interaction strengths in absence and presence of bridge protein FEP3 or FEP3-N (±Met) of panel **(B)** FIT-C-bHLH39, **(C)**, BTSL1-C-ILR3, **(D)** BTSL1-C-PYE, **(E)** BTSL1-C-bHLH34-C, **(F)** BTSL1-C-bHLH115-C, **(G)** BTSL1-C-bHLH104-C interactions. Yeast cells are grown in SD-LWM, for bridge protein expression and SD-LW for repression of bridge protein expression; ß- galactosidase activity is determined in Miller Units (MU). Data are represented as mean values with standard deviations. Different letters indicate statistically significant differences (one-way ANOVA and Tukey’s *post-hoc* test, *n* = 5, *p* < 0.05). Immunoblot analysis of FEP3/IMA1 is shown in Supplementary Figuree 13.

We tested the effect of FEP3/IMA1 on BTSL1-bHLH interactions using a quantitative yeast three hybrid (Y3H) assay. Y3H was designed to quantify ß-galactosidase activity as output of interaction between two proteins fused with AD or BD, in our case BTSL1 and a bHLH protein. Modulation of ß-galactosidase activity is quantified in the presence of an active vs. inactive so-called bridge protein, in our case FEP3/IMA1 vs. inactive FEP3-N. This assay allows testing whether the bridge protein has activating, repressing or neutral effect on the interaction complex formation of the AD/BD-fusion proteins (Figure 7A). By testing the effect of FEP3/IMA1 vs. FEP3-N on FIT and bHLH39 interaction, no difference was found. Instead, high b-galactosidase values indicated strong interaction of FIT and bHLH039 in all cases irrespective of FEP3/IMA1 or FEP3-N presence or absence (Figure 7B). This indicated that FEP3/IMA1 and FEP3-N expression had a neutral effect on the FIT-bHLH039 interaction. This was expected, based on the fact that neither bHLH039 nor FIT interacted with FEP3/IMA1. Interesting effects of FEP3/IMA1 were seen in the case of PYE, bHLH034-C and bHLH115-C, where the presence of active FEP3/IMA1 strongly impacted the protein interaction with BTSL1-C compared with the presence of inactive FEP3-N, while no difference was seen for the ILR3 interaction (Figures 7C–G, compare presence of FEP3 at ± Met with FEP3-N at ± Met). Additionally, the bridge protein is expressed under a methionine (Met)-repressible promoter, which allows modulating the level of FEP3/IMA1 protein in the Y3H system. We found, however, similar levels of FEP3/IMA1 protein under + and -Met in all cases (Supplementary Figuree 13). Despite that, a difference was seen between + and -Met for PYE, bHLH034-C and bHLH115-C supporting a negative effect of FEP3/IMA1. In the case of bHLH104-C no difference was seen between + and -Met. No difference occurred for BTSL1-C and ILR3 interaction in the case of FEP3/IMA1 or FEP3N under + and -Met, either, showing that the Met regulation of FEP3/IMA1 was not as reliable as comparison with inactive FEP3-N (Figure 7C). The BTSL1-C + ILR3 interaction was stronger than the other tested BTSL1-C + bHLH TF interactions. We suspected that because of the strong BTSL1-C + ILR3 interaction, FEP3/IMA1 is not able to interfere as an effector with BTSL1-C and ILR3 protein interaction.

Finally, we predicted structures of BTSL1-bHLH TF-IMA protein complexes using the Alphafold-multimer tool to find an explanation for the varying degrees of attenuation of protein interaction by FEP3/IMA1. Interestingly, these predictions agreed with the molecular docking model: FEP3/IMA1 was found to bind in close proximity to the MC site of BTSL1. Moreover, structural alignment showed that all IMA proteins were predicted to bind to BTSL1 at this same MC site interface (Supplementary Figuree 14A). Furthermore, we applied this tool to predict BTSL1-PYE, BTSL1-ILR3, BTSL1-bHLH115, and BTSL1-bHLH104 structures and aligned the predicted structures of BTSL1-bHLH-IMA1/FEP3 protein complexes (Supplementary Figuree 14). This theoretical approach suggests that bHLH proteins bind BTSL1 at two interfaces (Supplementary Figurees 14B–E). According to the models, a region at the N terminus of bHLH TFs binds BTSL1, designated as interface A. C-terminal regions of the TF models bind to a proximal region of the BTSL1 MC site, termed interface B. FEP3/IMA1, on the other side, only binds to interface B. PYE was found to weakly attach to BTSL1 at interface A. Instead, N terminal regions of ILR3, bHLH104, and bHLH115 were predicted to strongly attach to BTSL1 at interface A. This agrees with Y3H data. BTSL1-PYE but not BTSL1-ILR3 interaction was affected by FEP3/IMA1. PYE formed weak interaction with BTSL1 mainly via the interface B while ILR3 interacted via interfaces A and B. The C-terminal fragments bHLH104-C and bHLH115-C probably interacted weakly with BTSL1 since only interface B but not interface A was present. Thus, IMA1/FEP3 might attenuate specifically interactions that are not compensated by strong links between bHLH TFs and BTSL1 at interface A. The models also demonstrate that the bHLH factors bind to BTSL1 in an area with intrinsically disordered regions (Supplementary Figurees 14B–E).

Taken together, FEP3/IMA1 but not FEP3-N can modulate the interaction of BTSL1 and bHLH proteins. FEP3/IMA1 attenuates the interaction of BTSL1 with bHLH factors at binding interface B, provided that the interaction of BTSL1 and bHLH proteins is moderate to weak. This can be specified by binding sites at two interfaces of BTSL1-bHLH interactions.

## Discussion

Little structural information has been available on how Fe-regulatory proteins interact. A targeted Y2H screen revealed the novel protein interactome BTSL1-bHLH-FEP3/IMA1, confirming that –Fe-induced co-expressed gene clusters contain information about protein interaction complexes. FEP3/IMA1 targets via its C-terminal end a small region termed M-C site within the C-terminus of BTSL1. bHLH factors bind to BTSL1 in the vicinity of this site. FEP3/IMA1 attenuates protein interactions of PYE and bHLH IVc TFs with BTSL1. The similar phenotypes of FEP3/IMA1 overexpression and *btsl1 btsl2* loss of function support that FEP3/IMA1 is a small effector protein that inhibits the BTSL1-bHLH interaction and thereby promotes Fe uptake. Hence, our study uncovered a novel mechanism of action of FEP3/IMA1.

### FEP3/IMA1 Is an Effector Protein Acting on the Interaction of BTSL1 With bHLH TFs

The BTSL1-bHLH-FEP3/IMA1 interactome was uncovered in a screen, subsequently shown by targeted interaction assays combined with deletion mapping and computational docking to narrow down and confirm the protein interaction sites. Finally, quantitative protein interaction assays provided evidence about FEP3/IMA1 being a negative modulator of the BTSL1-bHLH TF interaction. The results suggest a framework for explaining structure-function relationships and a mechanism of FEP3/IMA1 action as an inhibitory protein acting upon BTSL1. FEP3/IMA1 was selective and modulated the strength of BTSL1-bHLH interactions, namely BTSL1-C-PYE, BTSL1-C-bHLH104-C, BTSL1-C-bHLH115-C, and BTSL1-C-bHLH34-C. In all these cases, FEP3/IMA1 caused repression of interaction strength, suggesting competition at the BTSL1 binding site with TF fragments, which was confirmed by theoretical prediction tools. In no case did we observe an increased strength of protein interaction in the presence of FEP3/IMA1, excluding cooperative binding effects that stimulate the interaction. Interestingly, only the weak to moderate protein interactions between BTSL1 and bHLH TFs could be altered by FEP3, but not the strong interactions, like BTSL1-C-ILR3. Clearly, the bHLH TFs differed in the number of interfaces for binding to BTSL1, whereby PYE-BTSL1 interaction appeared weaker with only interface B than that of ILR3-BTSL1 which relies on interfaces A and B. We interpret this finding to be very important in a biological context where differential interactions and their differing strengths are responsible to fine-tune Fe deficiency responses in balanced manner, depending on absence and presence of the various components of the interactome and differing binding sites. As discussed in the next paragraph the physiological data gained from FEP3/IMA1 overexpression and *btsl1 btsl2* mutants support that FEP3/IMA1 via BTSL1 targets the top of the –Fe bHLH response cascade. Since BTSL1 and BTSL2 are very similar in sequence and undergo similar protein interactions with FEP3/IMA1 and bHLH TFs, we predict that BTSL2 acts similar to BTSL1, but this requires further experiments. The related FEP1/IMA3 acts on BTS, however, the same sequence HVC is not conserved in BTS, although the PAA of the TFs were found important (Li et al., 2021), and also the M-C site of BTS shows multiple aa differences to the M-C site of BTSL1 and BTSL2. Moreover, PYE does not have the C-terminal PAA site as present in bHLH IVc TFs that interact via this motif with BTSL1. These findings together indicate that the protein interactions are far more complex and more structural details are needed to explain the various combinations and their effects. Because of the complexity of the BTSL1-bHLH-FEP3/IMA1 system, future studies need to address the competition effects and protein-ligand binding affinities and interaction strengths between all components of the BTS/BTSL, bHLH TFs and FEP/IMA system at the structural-biochemical level to decipher functional specificities of the responses.

### Molecular-Physiological Integration of the BTSL1-bHLH-FEP3/IMA1 Interactome

**FIGURE 8:**
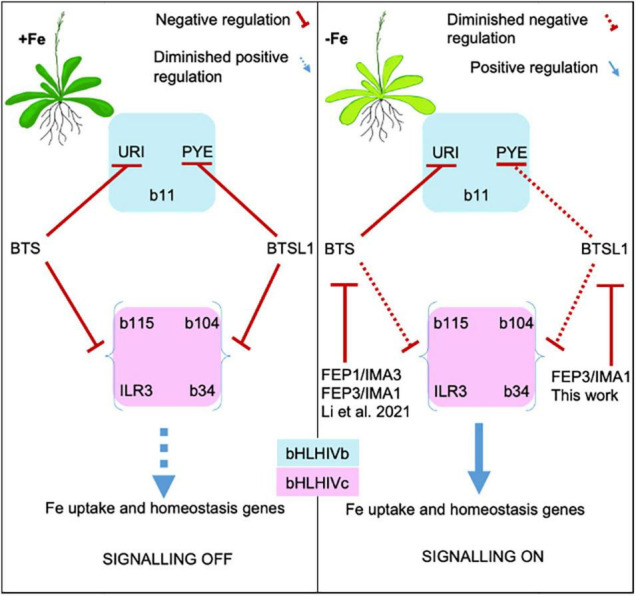
Model of FEP1/IMA3 and FEP3/IMA1 action to prevent BTS and BTSL1 protein-mediated degradation of bHLH factors of subgroups IVb and IVc. bHLH subgroup IVb and IVc TFs elicit Fe uptake and homeostasis in response to –Fe. These TFs are targets of E3 ligases BTS and BTSL1, possibly through degradation. BTS and BTSL1 target the same bHLH IVc but different bHLH IVb proteins. BTS, BTSL1, and the small effector proteins FEP1 and FEP3 are induced upon –Fe downstream of the bHLH IVc TFs. BTS and BTSL1 receive FEP1/FEP3 signals similar to receptor-ligand interactions. Binding of FEP1/FEP3 attenuates BTS/BTSL1-mediated degradation of bHLH TFs of subgroups IVb and IVc, allowing for enhanced Fe deficiency responses. Later, increased degradation of bHLH factors may halt Fe uptake. This model is based on intricate balancing of BTS/BTSL1-TF interaction strength and FEP1/FEP3 availability, allowing the cell to rapidly switch between on/off states to adjust Fe uptake and homeostasis. BTSL1-bHLH-FEP3/IMA1 interaction was shown in this work. BTSL2 interacts with FEP3/IMA1 and with similar TF proteins of the subgroup IVb and IVc as BTSL1, and may have a similar role as BTSL1 (this work). FEP1/IMA3- and FEP3/IMA1-BTS-bHLH interactions were shown by Li et al. (2021).

Physiological FEP3/IMA1 overexpression resembled loss of function of *btsl1 btsl2*, supporting the inhibitory effect of FEP3/IMA1 on BTSL1, observed in the Y3H assays. The gene expression profiles demonstrate that FEP3/IMA1 and BTSL1/BTSL2 act at the top level of the –Fe response cascade to affect the downstream target genes which comprised *BHLH* subgroup Ib genes, *PYE*, their co-expressed genes and further downstream targets of those. *BHLH* subgroup Ib and *PYE* genes are controlled by URI and bHLH IVc TFs. Among them, URI did not interact with FEP3/BTSL1/BTSL2, however, bHLH subgroup IVc TFs did. Therefore, BTSL1, presumably along with BTSL2, has a negative effect on bHLH IVc factors through interaction with them, attenuated by FEP3. *FEP3/IMA1* is normally co-expressed with *BHLH* subgroup Ib and *PYE* genes, however, this was not the case in *btsl1 btsl2*. Rodriguez-Celma et al. (2019) and Li et al. (2021) proposed that TFs of the subgroups IVb or IVc repress *FEP1/IMA3* gene expression, similar as rice homologs (Kobayashi et al., 2021). We suggest the following working model for the BTSL1-bHLH-FEP3/IMA1 interactome (Figure 8): BTSL1 interacts with PYE and bHLH IVc TFs to steer the top of a regulatory cascade leading to the Fe deficiency response in Arabidopsis. –Fe is sensed and bHLH IVc and URI TFs activate the –Fe response pathway in roots. *FEP3/IMA1* and *BTSL1* are up-regulated. BTSL1 protein binds PYE and bHLH IVc TFs, which downplays the –Fe response. In the presence of FEP3/IMA1, however, BTSL1 function is attenuated, allowing the TFs to be more active. Consequently, plants that constitutively up-regulate FEP3/IMA1 (in FEP3-Ox) or have no functional BTSL1 (in *btsl1 btsl2*) should accumulate Fe, which is what we observed, conform with previous studies of these mutants (Hindt et al., 2017; Grillet et al., 2018; Rodriguez-Celma et al., 2019; Li et al., 2021).

We initially struggled to explain why *ILR3* and *BHLH104* transcripts were down-regulated in FEP3-OX and *btsl1 btsl2* seedlings, although their downstream target genes were highly expressed. *ILR3*/*bHLH104* levels might also be controlled negatively by same TFs, possibly when the TFs are more active. This might be an additional layer of control to avoid excessive Fe uptake. This scenario actually could explain why neither *btsl1 btsl2*, nor *btsl1 btsl2 bts* triple mutants (Hindt et al., 2017) or FEP3-OX plants showed signs of severe Fe toxicity under +Fe. It was reported that PYE represses *ILR3* expression (Samira et al., 2018), hence *ILR3* down-regulation in FEP3-OX can be partly due to elevated PYE levels. In another study, ILR3 was shown to dimerize with PYE to repress *PYE* transcription (Tissot et al., 2019). Thus, bHLH IVc proteins in combination with PYE may control their own transcription. We also had expected that *PYE* and the downstream genes *NAS4*, *ZIF1*, and *FRO3* negatively regulated by PYE (Long et al., 2010) would have opposite expression patterns. However, although *PYE* was expressed at higher level in some conditions in mutant lines of this study, *NAS4* and *FRO3* were not down-regulated. This aligns with phenotypes of bHLH IVc gain-of-function lines (Zhang et al., 2015; Li et al., 2016), and indicates that PYE function can be bypassed or that bHLH IVc proteins and PYE act antagonistically to fine-tune Fe acquisition and internal Fe mobilization and allocation. Future studies need to address the network of gene regulation in plant lines with altered TF action in more detail.

This study also provided evidence that interaction of the BTSL1-bHLH system changes subcellular localization patterns. BTSL1 was mostly located at the cell periphery and only weakly in the nucleus. In contrast, BTS was located in the nucleus, as reported previously (Selote et al., 2015), except if the HHE domains were deleted, then the localization pattern shifted to the cytoplasm and was high in the root stele (Selote et al., 2015). Consistent with our results, the BTS homolog in rice, HRZ1, also localized to the nucleus while HRZ2 localized to nucleus and cytoplasm (Kobayashi et al., 2013). Interestingly, BTS and ILR3 localized and interacted in the nucleus, see also Selote et al. (2015). When BTSL1 was expressed together with PYE or ILR3 it was localized to the nucleus but still also at the cell periphery. This indicates that localization of BTSL1 is dependent on protein interaction partners in these experiments. PYE and ILR3 may be present in the cytoplasm or near the plasma membrane and in vicinity of plasmodesmata (Long et al., 2010; Selote et al., 2015). It might be conceivable that upon interaction with BTSL1, the entire complex shifts to the nucleus. The interaction of TFs with BTS and BTSL1 and hence their localization may depend on a combination of Fe availability and the presence of TFs. Interestingly, URI also has different localization in roots dependent on Fe supply (Gao et al., 2019a), and perhaps this pattern is also dependent on BTS. Alternatively, another factor may bind BTSL1 at the cell periphery. In this context, it is interesting to note that bHLH039 is present at the cell periphery when expressed alone, while the bHLH039-FIT complex is shifted to the nucleus (Trofimov et al., 2019). bHLH039 did not interact with BTSL1, so that it is unlikely that bHLH039 is the missing link for BTSL1 localization. The E3 ligases AtHOS1 (HIGH EXPRESSION OF OSMOTICALLY RESPONSIVE GENES1) shifts location from the cytoplasm into the nucleus during cold stress (Lee et al., 2001; Dong et al., 2006), and for AtRGLG1 (RING domain ligase 1) and AtRGLG2 it is the case upon abscisic acid or salt stress treatment (Cheng et al., 2012; Belda-Palazon et al., 2019). Because HOS1, RGLG1, and RGLG2 target nuclear proteins for degradation, the nuclear-localized E3 ligases could be the active forms during the stress conditions. FEP3/IMA1 was found distributed throughout the cell. However, we were not able to localize FEP3/IMA1 together with BTSL1. A reason might be that FEP3/IMA1 is degraded by BTSL1, in analogy to BTS that degrades FEP1/IMA3 and FEP3/IMA1 (Li et al., 2021). Taken together, future studies need to further address the localization of the interactome and its regulation by protein translocation inside the cell.

## Concluding Remarks

This study identified a protein interactome of bHLH subgroup IVc and PYE TFs with BTSL1 E3-ligase modulated by the small effector protein FEP3/IMA1. FEP3/IMA1 (<100 aa) is a small ORF-encoded protein (Delcourt et al., 2018), and such small proteins can act as ligands to receptors or by modulating protein-protein interactions (Makarewich and Olson, 2017). This and other studies could not find evidence for FEP3/IMA1 cleavage and secretion, excluding that it is a processed peptide hormone (Grillet et al., 2018; Hirayama et al., 2018). Instead, full-length HA-FEP3/IMA1 protein was detectable in our plants. Therefore, FEP3/IMA1 should be regarded a small effector protein rather than a peptide. Interestingly, small protein-E3 ligase interactions are known from animal systems. For example, the Drosophila *pri* interacts with the E3 ligase Ubr3, facilitating Ubr3 binding to the TF Svb. This changes Svb function (Zanet et al., 2015). From Drosophila as well as mammals, examples are known in which small proteins alter protein localization or bind to enzymes to affect their activity, either by direct competition with the substrate or in an allosteric manner (Cabrera-Quio et al., 2016). The localization and co-localization of BTSL1 in the presence and absence of TFs indicated patterns of regulation with regard to cellular partitioning of the protein interaction complexes in plant cells.

Several open questions will be of interest for future studies: A limitation in our study is that due to the lack of BTSL1 protein detection in plant cells, we were not able to fully validate the protein interactions in plants. Using precise deletions or substitutions of different predicted functional amino acids in BTSL1 may prevent degradation of BTSL1 in plant cells. Such an approach represents a promising solution provided that an altered three-dimensional protein structure does not hamper the protein interaction capability. BTSL1 and possibly also BTSL2 may ubiquitinate and degrade bHLH subgroup IVb and IVc TFs. FEP3/IMA1 homologs, that bind BTS, may also target BTSL1 and BTSL2. This allows space for an intricate control through balanced combinations of interactions between BTS/L, bHLH, and FEP/IMA proteins. Biochemical information as to the actual structural requirements, affinities and concentrations of players and their post-translational modifications might resolve functionality of the interactomes. These factors add an unprecedented layer of complexity to the negative regulation by the FEP/IMA effector mechanism. Certainly, the interactions of BTS/L, bHLH and FEP proteins diverged from a common ancestor interaction up to the level of diversity seen in higher plants today. The complexity arising from combination possibilities between all bHLH-BTS/L and FEP/IMA proteins may allow higher land plants to adequately adjust the action of the TFs in a multitude of developmental and physiological situations. Perhaps this double negative control was driven by evolutionary constraints in response to a changing environment of the plants. Identification of interaction sites within E3 ligases offers possibilities to engineer crops with modified bHLH IVb and IVc, E3 ligase or FEP/IMA binding sites. Indeed, several of the bHLH transcription factors we studied here have roles in abiotic stress protection in plants, e.g., in photoprotection (Akmakjian et al., 2021). Mechanistic understanding of bHLH subgroup IVb and IVc factors will therefore have broad impact to adapt plants to changing climate and to unravel the ecological significance of Fe usage efficiency during climate change.

## Materials and Methods

### Plant Material

Arabidopsis (*Arabidopsis thaliana*) ecotype Columbia-0 (Col-0) was used as wild type (WT) and as background for transgenic lines. Primers used are listed in Supplementary Table 2. The *btsl1 btsl2* loss-of-function double mutant (*btsl1-1 btsl2-2*, crossed SALK_015054 and SALK_048470) was described previously (Rodriguez-Celma et al., 2019). T-DNA insertion sites were verified with primer pairs LBb1.3/btsl1-1_RP (*btsl1*) and LBb1.3/btsl2-2_RP (*btsl2*) and homozygosity was verified with the primer pairs btsl1-1_LP/btsl1-1_RP and btsl2-2_LP/btsl2-2_RP (Supplementary Figure 9). For plant lines ectopically over-expressing triple HA-tagged *FEP3/IMA1* (FEP3-OX) under the control of a double CaMV 35S promoter, the coding sequence (CDS) was amplified from cDNA of Arabidopsis WT roots with primers carrying B1 and B2 attachment sites, respectively, transferred into the entry vector pDONR207 (Invitrogen) according to the manufacturer’s recommendations (BP reaction, Gateway, Thermo Fisher Scientific, Life Technologies GmbH, Darmstadt, Germany). Final constructs were obtained by transferring all candidate genes subsequently into the plant binary destination vector pAlligator2 (N-terminal triple HA fusions = HA_3_) (Bensmihen et al., 2004) via LR reactions (Thermo Fisher Scientific). Constructs were transformed into Agrobacteria (*Rhizobium radiobacter*) strain GV3101 (pMP90). Stable transgenic Arabidopsis lines were generated via the Agrobacterium-mediated floral dip method (Clough and Bent, 1998). Positive transformants were selected based on seed GFP expression and genotyping PCR on the transgenic cassette, selfed and propagated to T3 generation. Insertion sites of the transgenic cassettes in FEP3-OX#1 and FEP3-OX#3 were determined by thermal asymmetric interlaced (TAIL) PCR with the primers S1_AL2_LB (template: gDNA), S2_AL2_LB (template: S1_AL2_LB amplicon), S3_AL2_LB (template: S2_AL2_LB amplicon), each combined with AD1, AD2, AD3, AD4, AD5, AD6. The insertion sites were verified by genotyping PCR with primer pairs FEP3-OX1_chr5 fw/S3_AL2_LB (FEP3-OX#1), FEP3-OX3_chr1 fw/S3_AL2_LB (FEP3-OX#3). Homozygosity was determined with primer pairs FEP3-OX1_chr5 fw/FEP3-OX1_chr5 rev (FEP3-OX#1), FEP3-OX3_chr1 fw/FEP3-OX3_chr1 rev (FEP3-OX#3). Promoter sequences of *BTS* (2,994 bp), *BTSL1* (880 bp), *PYE* (1,120) and *FEP3/IMA1* (1,614 bp) were amplified from Arabidopsis WT gDNA with primer pairs proBTS_-2994_B1 fw/proBTS_-2994_B2 rev (for *proBTS*), proBTSL1_-880_B1 fw/proBTSL1_-880_B2 rev (for *proBTSL1*), proPYE_-1120_B1 fw/proPYE_-1120_B2 rev (for *proPYE*), proFEP3_-1614_B1 fw/proFEP3_-1614_B2 rev (for *proFEP3*) and proFEP3_-1614_B1 fw/FEP3ns_B2 rev (for *proFEP3:FEP3*), respectively, cloned into pDONR207 (Invitrogen). Sequences were transferred into the vector pGWB3 (Nakagawa et al., 2007), generating *proBTS:GUS*, *proBTSL1:GUS*, *proPYE:GUS*, *proFEP3:GUS*, and *proFEP3:FEP3-GUS* constructs. Constructs were transformed into Arabidopsis WT plants as described above (Clough and Bent, 1998). Positive transformants were selected based on hygromycin resistance and genotyping PCR, selfed and propagated to T2 or T3 generation. *ProILR3:GUS*/WT and *proBHLH104:GUS*/WT Arabidopsis lines were described (Li et al., 2016).

### Plant Growth Conditions

Arabidopsis seeds were surface-sterilized and stratified. For experimental analyses seeds were distributed to sterile plates containing modified half-strength Hoagland medium [1.5 mM Ca(NO_3_)_2_, 1.25 mM KNO_3_, 0.75 mM MgSO_4_, 0.5 mM KH_2_PO_4_, 50 μM KCl, 50 μM H_3_BO_3_, 10 μM MnSO_4_, 2 μM ZnSO_4_, 1.5 μM CuSO_4_, 0.075 μM (NH_4_)_6_Mo_7_O_24_, 1% (w/v) sucrose, pH 5.8, and 1.4% w/v plant agar (Duchefa)] with (Fe sufficient, +Fe) or without (Fe deficient, –Fe) 50 μM FeNaEDTA and vertically grown in plant growth chambers (CLF Plant Climatics, Wertingen, Germany) under long day conditions (16 h light/8 h dark), as described in Lingam et al. (2011). Seedlings were grown for six or ten days directly on +Fe or –Fe medium [6 day (d) system/10 d system, 6-day-old/10-day-old seedlings exposed to ±Fe] (Lingam et al., 2011). Alternatively, seedlings were grown for 14 days on +Fe medium and then transferred for 3 days to either +Fe or –Fe (14 + 3 d system, 14-day-old plants exposed to ±Fe), as indicated in the text.

### Yeast Assays

#### Targeted Yeast Two Hybrid Screen

Twenty three protein interactions were tested using N-terminal AD (pACT2-GW constructs) and BD (pGBKT7-GW constructs) fusion proteins (vectors from Clontech, Takara Bio Europe SAS, Saint-Germain-en-Laye, France). If possible, interactions were studied in both “reciprocal” combinations AD/BD and BD/AD. The interaction was considered more robust when detected in reciprocal manner than in only one direction, however, some proteins could not be tested in both situations, either because of auto-activation or steric hindrance. For Y2H assays, CDS were amplified from cDNA of Arabidopsis WT roots with primers carrying B1 and B2 attachment sites (Supplementary Table 2), respectively and transferred into pDONR207 (Thermo Fisher Scientific, Darmstadt, Germany). Finally, all candidate genes were transferred into destination vectors pACT2-GW and pGBKT7-GW. Yeast (*Saccharomyces cerevisiae*) strain Y187 was transformed with pACT2-GW (AD) constructs and yeast strain AH109 with pGBKT7-GW (BD) constructs via the lithium acetate (LiAc) method, based on (Gietz and Schiestl, 2007). Transformants were selected by cultivation for 2 days on minimal synthetic defined (SD) media Clontech (Takara Bio Europe SAS, Saint-Germain-en-Laye, France) lacking Leu (pACT2-GW) or Trp (pGBKT7-GW). Yeast expressing both *AD* and *BD* constructs were obtained by mating and selected on minimal SD media lacking Leu and Trp (SD-LW). To test for protein-protein interaction, a fresh diploid colony was resuspended in sterile H_2_O to OD_600_ = 1 and 10 μl of the suspensions were dropped onto minimal SD media lacking Leu, Trp and His (SD-LWH), containing appropriate concentrations of 3-amino-1,2,4-triazole (3-AT). It was necessary to adjust 3-AT concentrations individually to obtain reliable and valid interaction data while avoiding auto-activation of the BD fusion proteins. Plates were cultivated at 30°C for up to 14 days. Diploid cells expressing each pACT2-GW:*X* construct in combination with an empty pGBKT7-GW and vice versa were used as negative controls. Combination of pGBT9.BS:*CIPK23* and pGAD.GH:*cAKT1* was used as a positive control of the system, FIT-C was used as it is not self-activating in the assay (Gratz et al., 2020).

#### Targeted Yeast Two Hybrid Assays for Validation

Selected protein pairs of the Y2H screen plus additional proteins (URI, bHLH11, bHLH34, bHLH115) and mutagenized/truncated protein versions were assayed as N-terminal AD and BD fusion proteins in both reciprocal combinations as detailed above. Mutagenized *BTSL1* versions *BTSL1-dRH*, *BTSL1-6G*, and *BTSL1-dMC* were created as described in “BTSL1 mutagenesis.” Truncated versions *BTSL1-N*, *BTSL1-C*, *BTSL1-C.1*, *BTSL1-C.2*, *BTSL1-C.3*, *BTSL1-C.4*, *FEP3-N*, *FEP3-C*, *FEP3-d7*, *ILR3-d25*, *ILR3-CC*, *bHLH104-C*, *bHLH104-C-d25*, *bHLH104-CC* were amplified with primers listed in Supplementary Table 2 and cloned into pACT2-GW and pGBKT7-GW as described in the previous section. Yeast strain AH109 was co-transformed with both pACT2-GW:*X* (AD-X) and pGBKT7-GW:*Y* (BD-Y) (including empty vector controls) as described in the previous section. X and Y represent proteins of a tested protein pair. Haploid double transformants were selected on minimal SD media lacking Leu and Trp. To select for protein-protein interaction, overnight liquid cultures were adjusted to OD_600_ = 1 and dilution series down to OD_600_ = 10^–4^ were prepared. 10 μl of the suspensions were dropped onto SD media lacking Leu, Trp and His and containing the appropriate 3-AT concentration and cultivated as described in the previous section.

#### Yeast Three-Hybrid Assays

Genes which code for bridge protein were transferred from pDONR207 into pBRIDGE-GW using Gateway technology (Thermo Fisher Scientific, Darmstadt, Germany). Genes which code for bait proteins were cloned adjacent to Gal4-BD sequence using AQUA cloning method (Beyer et al., 2015). Prey protein constructs were prepared in pACT2-GW vector as mentioned previously. Primers are listed in Supplementary Table 2. Yeast (*Saccharomyces cerevisiae*) strain Y190 was transformed with pACT2-GW (AD) constructs and pBRIDGE-GW (BD-Bridge) constructs via the lithium acetate (LiAc) method, based on (Gietz and Schiestl, 2007). Co-transformants were selected by cultivation for 2 days on minimal synthetic defined (SD) media (Clontech) lacking Leu (pACT2-GW) or Trp (pBRIDGE-GW). Beta(β)-Galactosidase assay was performed using Yeast β-Galactosidase Assay Kit (Thermo Fisher Scientific, Darmstadt, Germany), with ortho-Nitrophenyl-β-galactoside (ONPG) as substrate. Freshly grown co-transformants in SD-LT and SD-LTM were used in the assay to extract enzyme. Initially OD 600 of the cultures was measured and cells pelleted. Yeast proteins were extracted, and beta-Galactosidase assay solution was added to extract, mixed and incubated for 30 min to 3 h. Absorbance at 420 nm was measured using the Infinite 200 Pro microplate reader, TECAN. ß-galactosidase activity was calculated using Miller‘s formula, in Miller units (MU) ß-galactosidase activity = (1,000 * Absorbance 420)/(O.D 660* t* V); t = time in minutes of incubation, V = volume of cells used in the assay. The presence of the bridge protein HA-FEP was detected by anti-HA immunoblot analysis. Yeast proteins were harvested by agitating cells in Y-PER Yeast Protein Extraction Reagent (Thermo Fisher Scientific, Darmstadt, Germany). Equal amounts of total protein were separated on SDS-polyacrylamide gels, and transferred to a Protran nitrocellulose membrane. The membrane was blocked with 5% (w/v) milk solution in 1xTBST [150 mM NaCl, 2.7 mM KCl, 24.7 mM Tris-HCl, 0.1% (v/v) Tween 20, pH 7.4] for 30 min and subsequently incubated 1 h with anti-HA-peroxidase high-affinity monoclonal rat antibody (3F10; Roche Holding AG, Basel, Switzerland [catalog no. 12013819001]) diluted 1:1,000 in 2.5% (w/v) milk solution. After three wash steps, each for 15 min in TBST, the membrane was imaged as described in Le et al. (2016). Chemiluminescent protein bands were detected with the FluorChem Q system (ProteinSimple, San Jose, CA, United State) and images were processed with the AlphaView software (version 3.4.0.0, ProteinSimple, San Jose, CA, United State).

### Histochemical β-Glucuronidase Assay

Seedlings were analyzed for β-glucuronidase (GUS) activity using 2 mM 5-bromo-4-chloro-3-indoyl-b-D-glucuronic acid (X-Gluc) as substrate and incubated at 37°C in the dark for 15 min up to 12 h. From *proBTSL1:GUS, proFEP3:GUS* and *proFEP3:FEP3-GUS* lines, four to six seedlings were fixed in ice cold 90% acetone for 1 h and washed in phosphate buffer prior to incubation in the GUS staining solution, which was vacuum infiltrated to obtain better staining. Incubation was performed as described above and stained tissue was fixed in 75% ethanol and 25% acetic acid for 2 h at RT. Chlorophyll was removed by incubation in 70% ethanol for 24 h. Seedlings were imaged with the Axio Imager M2 (Carl Zeiss AG, Oberkochen, Germany, 10× objective magnification) and images of entire seedlings assembled with the Stitching function of the ZEN 2 BLUE Edition software (Carl Zeiss AG, Oberkochen, Germany).

### Subcellular (Co-) Localization

To observe subcellular localization, proteins were tagged C-terminally to GFP and/or mCherry fluorophores and/or N-terminally to YFP fluorophore and expressed transiently in tobacco leaf epidermal cells via Agrobacterium-mediated leaf infiltration. For N- and C-terminal fusions, CDSs were amplified from cDNA of Fe Arabidopsis WT roots with primers carrying B1 and B2 attachment sites (Supplementary Table 2), transferred into the entry vector pDONR207 (Thermo Fisher Scientific, Darmstadt, Germany) and subcloned into destination vectors pMDC83 (C-terminal GFP fusions) (Curtis and Grossniklaus, 2003), pH7WGY2 (N-terminal YFP) (Karimi et al., 2005), and β-estradiol-inducible pABind-GFP and pABind-mCherry (C-terminal GFP and mCherry, used in co-localization studies) (Bleckmann et al., 2010). The constructs were transformed into Agrobacteria as described in “Plant Material”. A suspension (OD_600_ = 0.4) of Agrobacteria carrying the construct of interest in infiltration solution [2 mM NaH_2_PO_4_, 0.5% (w/v) glucose, 50 mM MES, 100 μM acetosyringone (in DMSO), pH 5.6] was infiltrated into tobacco leaves using a 1 ml syringe pressed to the abaxial leaf side. For co-localization corresponding Agrobacteria suspensions were mixed 1:1 (each to an OD_600_ = 0.4) prior to infiltration. For more efficient expression, Agrobacteria carrying the p19 plasmid were co-infiltrated (suppression of RNA interference) (Voinnet et al., 2003, 2015). Transformed plants were kept at RT under long day conditions (16 h light, 8 h dark) and imaged after 48–72 h with a LSM 510 meta confocal laser scanning microscope (Carl Zeiss AG, Oberkochen, Germany) or an Axio Imager M2 with ApoTome (Carl Zeiss AG, Oberkochen, Germany). GFP and YFP were imaged at an excitation wavelength of 488 nm and emission wavelength of 500–530 nm, mCherry was imaged at an excitation wavelength at 563 nm and emission wavelength of 560–615 nm. Expression of pABind constructs was induced by spraying β-estradiol mix [20 μM β-estradiol (in DMSO), 0.1% (v/v) Tween20] to the abaxial leaf side 24–48 h post-infiltration (24–48 h before imaging). The (co-) localization experiments were performed in at least two independent replicates or as indicated in the text. Plasmolysis of cells expressing *BTSL1-GFP* was achieved through treatment of the leaf sample with 1 M mannitol solution for 30 min.

### Bimolecular Fluorescence Complementation

CDS of gene pairs to be tested were amplified from cDNA of Arabidopsis WT roots. Amplicons generated with primers carrying B3 and B2 attachment sites were transferred into pDONR221-P3P2 (Thermo Fisher Scientific, Darmstadt, Germany, for nYFP fusion) and amplicons generated with primers carrying B1 and B4 attachment sites were transferred into pDONR221-P1P4 (Thermo Fisher Scientific, Darmstadt, Germany, for cYFP fusion), respectively. Primer sequences are listed in Supplementary Table 2. In a multisite Gateway LR reaction (Thermo Fisher Scientific, Darmstadt, Germany), both genes were transferred simultaneously into destination vector pBiFCt-2in1-NN (N-terminal nYFP and cYFP fusions) (Grefen and Blatt, 2012), to create pBiFCt-2in1-NN: FEP3:BTSL1, pBiFCt-2in1-NN:PYE-BTSL1, pBiFCt-2in1-NN:PYE-BTSL1-C and pBiFCt-2in1-NN:ILR3-BTSL1-C. The constructs carry a monomeric red fluorescent protein (mRFP) as internal transformation control. As negative controls, structurally similar proteins known to not interact were used (negative controls: pBiFCt-2in1-NN:ILR3-BTSL2-C, pBiFCt-2in1-NN:FIT-BTSL1-C) (Kudla and Bock, 2016). Constructs were transformed into Agrobacteria and subsequently infiltrated into tobacco leaves, as described above. Forty 8–52 h after infiltration, mRFP and YFP signals were detected with an Axio Imager M2 (Carl Zeiss AG, Oberkochen, Germany). YFP was imaged at an excitation wavelength of 488 nm and emission wavelength of 500–530 nm, mRFP was imaged at an excitation wavelength at 563 nm and emission wavelength of 560–615 nm. BiFC experiments were performed in at least two independent replicates with two infiltrated leaves each.

### Gene Expression Analysis by RT-qPCR

Gene expression analysis was performed as described earlier (Abdallah and Bauer, 2016). In brief, mRNA was extracted from whole seedlings grown in the 6 d system (*n* > 60 per replicate) or from roots grown in the 14 + 3 d system (*n* > 15 per replicate) (see “Plant Growth Conditions”) and used for cDNA synthesis. RT-qPCR was performed using the iTaq™ Universal SYBR^®^ Green Supermix (Bio-Rad Laboratories, Hercules, CA, United States) and the SFX96 Touch™ RealTime PCR Detection System (Bio-Rad Laboratories, Hercules, CA, United States). Data was processed with the Bio-Rad SFX Manager™ software (version 3.1). Absolute gene expression values were calculated from a gene specific mass standard dilution series and normalized to the elongation factor *EF1B*α. Primers for mass standards and RT-qPCR are listed in Supplementary Table 2. The analysis was performed with three biological and two technical replicates.

### Immunoblot Analysis

Total proteins were extracted from ground plant material (tobacco leaves or Arabidopsis whole seedlings grown in the 6 d system, n = 30-60 seedlings) with 2× Laemmli buffer [124 mM Tris-HCl, pH 6.8, 5% (w/v) SDS, 4% (w/v) dithithreitol, 20% (v/v) glycerol, with 0.002% (w/v) bromophenol blue] and denatured at 95°C for 10 min. Equal amounts of total protein were separated on SDS-polyacrylamide gels, transferred to a Protran nitrocellulose membrane and stained with PonceauS as described in Le et al. (2016). To detect HA_3_-tagged FEP3/IMA1 protein, the membrane was blocked with 5% (w/v) milk solution in 1xPBST (137 mM NaCl, 2.7 mM KCl, 10.14 mM Na_2_HPO_4_, 1.76 mM KH_2_PO_4_, 0.1% (v/v) Tween^®^ 20, pH 7.4) for 30 min and subsequently incubated 1 h with anti-HA-peroxidase antibody and detected as described in Yeast Three-Hybrid assays.

### Root Length Measurement

Plants were photographed at day six. Length of primary roots of individual seedlings was measured using the JMicroVision software (version 1.2.7),^1^ as described previously (Ivanov et al., 2014). For calculation of mean root lengths and standard deviations, *n* = 13–29 roots per line and condition were measured.

### Seed Fe Content Measurement

To determine seed Fe content, 1–3 plants from each line were grown on soil under long day conditions (16 h light, 8 h dark, 21°C). Seeds were harvested, pooled by plant genotype, and dried for 16 h at 100°C. Fe was extracted from ground seed material by incubation in 500 μl 3% (v/v) HNO_3_ for 16 h at 100°C. Fe content in the supernatant was determined as described (Tamarit et al., 2006). Total Fe content in the sample was calculated with the help of a standard curve and normalized to seed dry weight. Per seed pool, *n* = 3 samples were measured.

### Multiple Sequence Alignments and Protein Sequence Conservation

Multiple sequence alignments were performed with ClustalX using default settings (Larkin et al., 2007). To determine conservation scores of aa in BTSL1, the full BTSL1 aa sequence was uploaded to the Basic Local Alignment Search Tool [BLAST, Altschul et al. (1990)^2^ ] and run against the Viridiplantae database using the standard blastp (protein-protein BLAST) algorithm. The top 100 hits were downloaded, duplicates were removed. The remaining sequences were used for multiple sequence alignment using the Clustal Omega algorithm (Sievers et al., 2011) and visualized with Jalview (Waterhouse et al., 2009).^3^ The full aa sequence of FEP3/IMA1 run against the Viridiplantae database as described above. Hits were only found within the Brassicaceae family, but alignments showed sequence conservation specifically toward the C-terminus. Subsequent blastp of the C-terminal half of FEP3/IMA1 (25 aa) resulted in several angiosperm hits. FEP3/IMA1 ortholog sequence hits from exemplary angiosperm orders were downloaded and aligned.

### Protein Structure Prediction and Molecular Docking

Protein structures were predicted using AlphaFold2 (Jumper et al., 2021) the Alphafold-Multimer tool (Evans et al., 2021) with protein sequences from TAIR. Multiple Sequence alignments were generated through MMseqs2 API. Molecular docking was performed in HADDOCK 2.4 (van Zundert et al., 2016; Honorato et al., 2021). Active residues were used to generate ambiguous interaction restraints. The obtained file was further processed in Discovery studio, Dassault Systems BIOVIA and UCSF Chimera.

### Statistical Analysis

Null hypothesis between normally distributed groups was tested with a two-tailed Student’s *t*-test. Null hypothesis was rejected, when the *p*-value (*p*) was below 0.05. Statistically significantly different groups are indicated by one asterisk for *p* < 0.05, two asterisks for *p* < 0.01 and three asterisks for *p* < 0.001. When comparing more than two groups, null hypotheses were tested with one-way analysis of variance (ANOVA) and a Tukey’s *post-hoc* test. Null hypotheses were rejected when *p* < 0.05. Statistically significantly different groups are indicated by different lower-case letters. Number of technical and biological repetitions of the individual experiments are indicated in the Figure legends.

## Accession Numbers

AKT1 (AT2G26650), BHLH11 (AT4G36060), BHLH34 (AT3G23210), BHLH38 (AT3G56970), BHLH39 (AT3G56980), BHLH100 (AT2G41240), BHLH101 (AT5G04150), BHLH104 (AT4G14410), BHLH115 (AT1G51070), BTS (AT3G18290), BTSL1 (AT1G74770), BTSL2 (AT1G18910), CIPK23 (AT1G30270), DGAT3 (AT1G48300), DUF506 (AT1G12030), FEP1 (AT2G30766), FEP3/IMA1 (AT1G47400), FIT (AT2G28160), FRO2 (AT1G01580), FRO3 (AT1G23020), GRF11 (AT1G34760), ILR3 (AT5G54680), IRT1 (AT4G19690), JAL12 (AT1G52120), KELCH (AT3G07720), MYB72 (AT1G56160), NAS2 (AT5G56080), NAS4 (AT1G56430), ORG1 (AT5G53450), PRS2 (AT1G32380), PYE (AT3G47640), SDI1 (AT5G48850), S8H (AT3G12900), TCP20 (AT3G27010), UP1 (AT3G06890), UP2 (AT3G56360), UP3 (AT5G05250), and URI (AT3G19860).

## Data Availability Statement

The original contributions presented in this study are included in the article/Supplementary Material, further inquiries can be directed to the corresponding author.

## Author Contributions

DL, BS, DB, and PB conceived the project and reviewed and edited the manuscript. DL, BS, DB, CE, and CS performed the experiments and analyzed the data. DL, BS, and PB supervised the research. BS and DL wrote the original draft. PB acquired funding and agreed to serve as the author responsible for contact and ensures communication. All authors contributed to the article and approved the submitted version.

## Conflict of Interest

The authors declare that the research was conducted in the absence of any commercial or financial relationships that could be construed as a potential conflict of interest.

## Publisher’s Note

All claims expressed in this article are solely those of the authors and do not necessarily represent those of their affiliated organizations, or those of the publisher, the editors and the reviewers. Any product that may be evaluated in this article, or claim that may be made by its manufacturer, is not guaranteed or endorsed by the publisher.

## Footnotes

1
http://www.jmicrovision.com

2
https://blast.ncbi.nlm.nih.gov/

3
http://www.jalview.org/

## Abbreviations

3AT: 3-amino-1,2,4-triazole
aa: amino acid
AD: activation domain
BD: binding domain
bHLH: basic helix-loop-helix
BiFC: bimolecular fluorescence complementation
GFP: green fluorescent protein
GUS: β-glucuronidase
HHE: hemerythrin/HHE cation-binding motif
mCherry: second generation mRFP derivative
mRFP: monomeric red fluorescent protein
ORF: open reading frame
OX: over-expression
RT-qPCR: reverse transcription quantitative PCR
SD: standard deviation (Statistics)/synthetic defined medium (Y2H)
TF: transcription factor
WT: wild type
Y2H: yeast two-hybrid
Y3H: yeast three-hybrid
YFP: yellow fluorescent protein.
