# Supplementary material for: The Iron Deficiency-Regulated Small Protein Effector FEP3/IRON MAN1 Modulates Interaction of BRUTUS-LIKE1 With bHLH Subgroup IVc and POPEYE Transcription Factors
Source: Front Plant Sci. 2022 Jun 10;13:930049. doi: 10.3389/fpls.2022.930049 (PMC9226616; doi:10.3389/fpls.2022.930049)
Supplement: Supplementary file 1 [file Data_Sheet_1.pdf]

Supplementary Figure 1

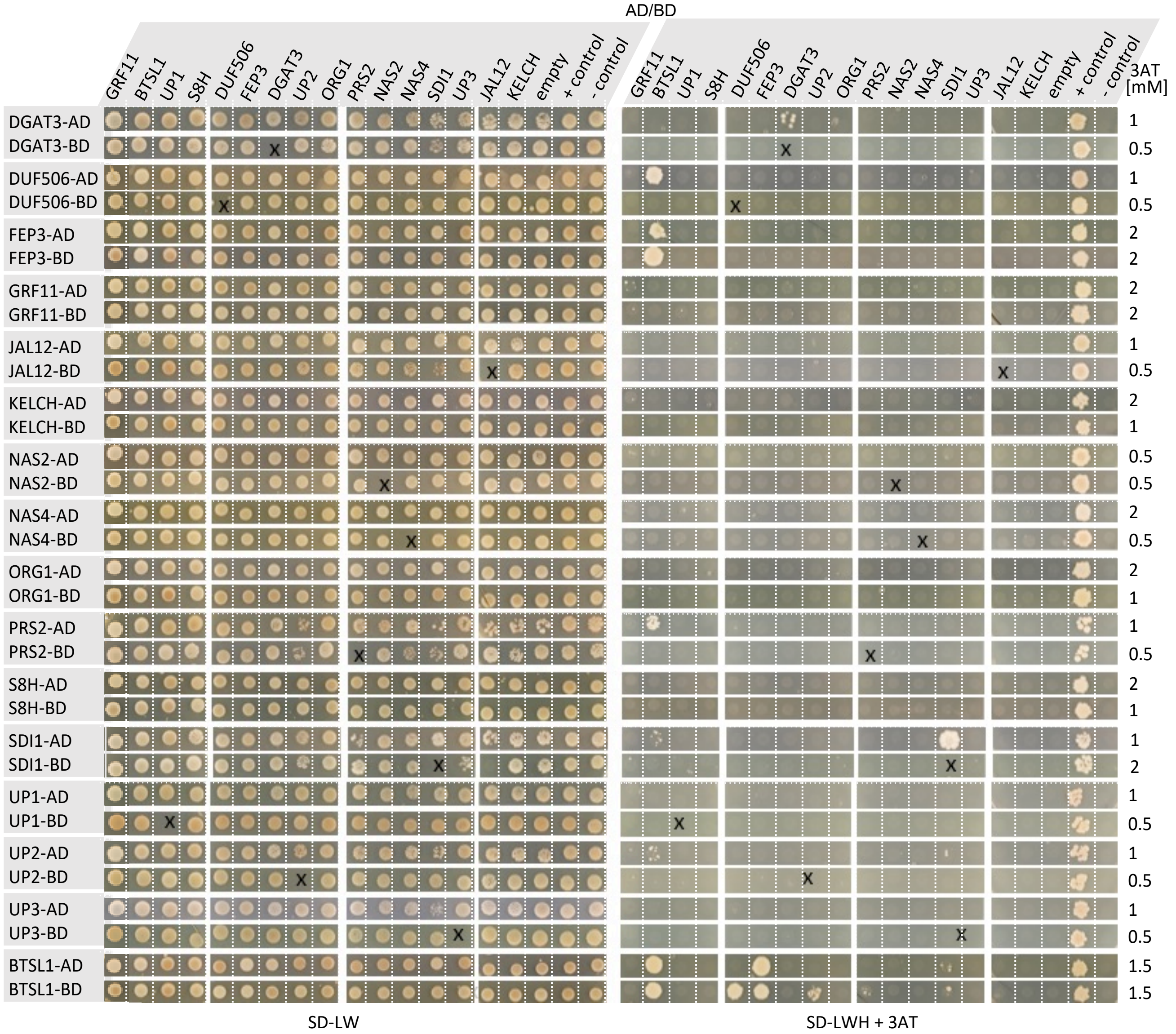

Supplementary Figure 1: Targeted Y2H screen (continued in Supplementary Figure 2).

Targeted Y2H screen between 16 out of 23 candidates (non-TFs) in pair-wise reciprocal combinations including homodimerization. Yeast containing both AD- and BD-plasmids were obtained by mating of single transformants and spotted as A600=1 on SD-LW (control condition) and SD-LWH supplemented with different concentrations of 3-AT (3AT) as indicated on the top right side (selection condition). It was necessary to adjust 3-AT concentrations individually to obtain reliable and valid interaction data while avoiding auto-activation of the BD fusion proteins. Positive control (+ control), CIPK23/cAKT1; negative controls (empty), AD-protein/empty and BD-protein/empty. X, evaluation not possible because of control.

Supplementary Figure 2

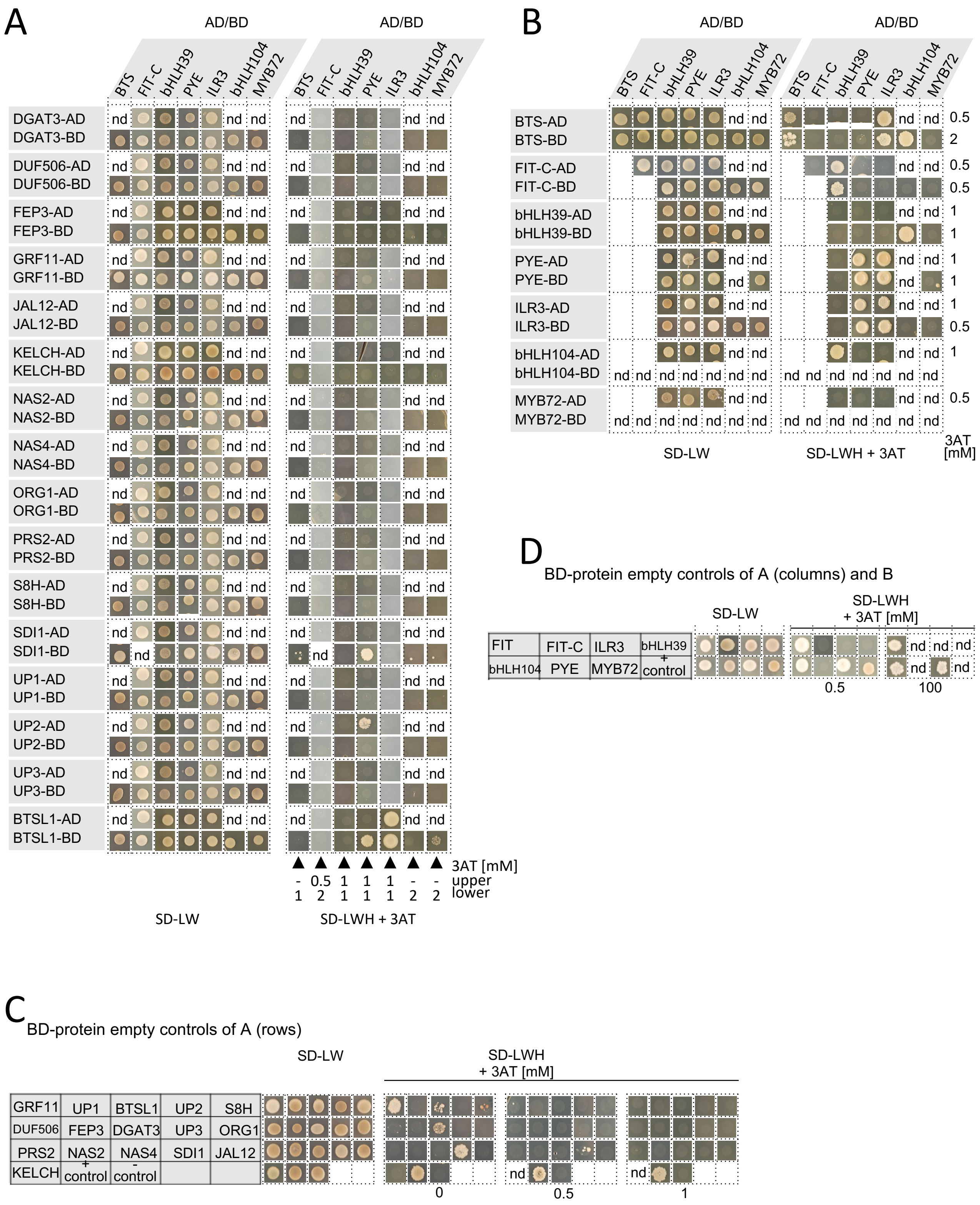

Supplementary Figure 2. Targeted Y2H screen (continued).

A, Targeted Y2H screen between the 16 candidates shown in Supplementary Figure 1 (rows) and 7 out of 23 candidates (6 TFs and BTS) in pair-wise reciprocal combinations. Yeast containing both AD- and BD-plasmids were obtained by mating of single transformants and spotted as A600=1 on SD-LW (control) and SD-LWH supplemented with different concentrations of 3AT as indicated on the bottom right side (selection). Positive control (+ control), CIPK23/cAKT1 in Supplementary Figure 1; negative controls (empty), AD-protein/empty and BD-protein/empty. B, Targeted Y2H screen between 6 TFs and BTS in pair-wise reciprocal combinations, including homodimerization. Except BTS and FIT-C, all protein pairs appear twice in the matrix. Interaction between AD-bHLH104/BD-bHLH39 in B. Labeling as in Supplemental Figure S1. C, Determination of 3AT concentrations necessary to suppress background activity for 16 non-TF BD-fusions (= BD protein/empty negative controls). Positions of protein names in the table correspond to yeast spot positions in the images. Negative controls correspond to the 16 BD proteins in A (rows). It was necessary to adjust 3-AT concentrations individually to obtain reliable and valid interaction data while avoiding auto-activation of the BD fusion proteins. Positive control (+ control), CIPK23/cAKT1; negative control (- control), empty/empty. D, Determination of 3AT concentration to suppress background activity for 7 TF/BTS BD-fusions (= BD protein/empty negative controls). Negative controls correspond to the 6 BD-proteins in A (columns) and in B (rows and columns), and of full-length FIT. Controls as described in C.

FIT-C, C-terminal part of FIT; nd, no data available. BD-bHLH104 and BD-MYB could not be tested because of self-activation.

Supplementary Figure 3

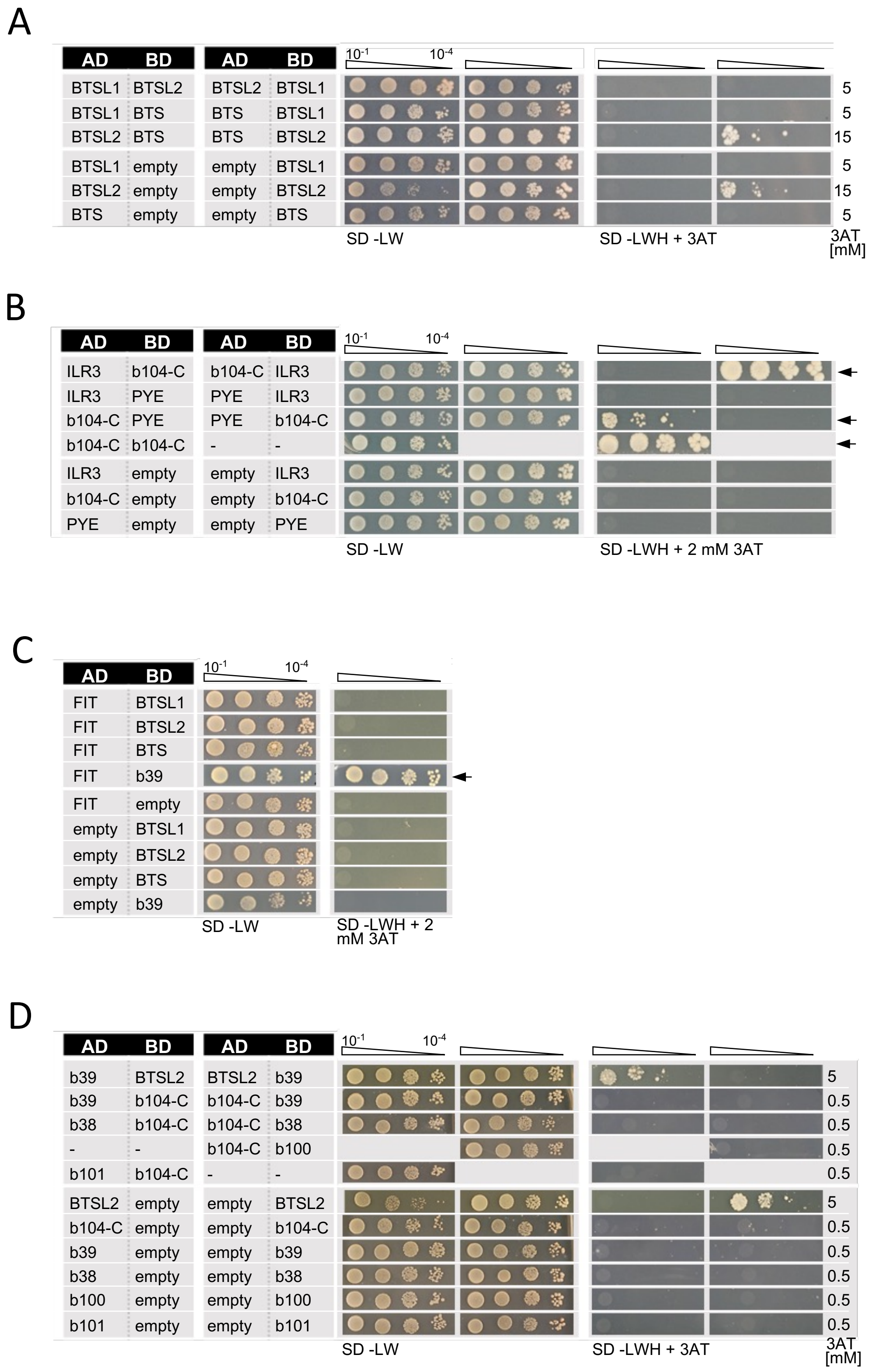

Supplementary Figure 3. Validation of protein interactions

A, Interaction study between BTSL1, BTSL2, BTS. B, Interaction study between ILR3, bHLH104-C, PYE, homodimerization of bHLH104-C. C, Interaction study between FIT and BTS/BTSL1/BTSL2. FIT+bHLH039 (b39), positive control (arrow). BD-FIT, not tested because of auto-activation (see Supplementary Figure 2D). D, Interaction study between bHLH subgroup Ib and selected subgroup IVc proteins and BTSL2.

Yeast co-transformed with the AD and BD combinations were spotted in 10-fold dilution series (A_{600}=10^{-1}-10^{-4}) on SD-LW (transformation control) and SD-LWH plates supplemented with different concentrations of 3AT, as indicated on the right side (selection for protein interaction). It was necessary to adjust 3-AT concentrations individually to obtain reliable and valid interaction data while avoiding auto-activation of the BD fusion proteins. b, bHLH; -C, C-terminal part used; negative controls, empty vectors; arrows indicate interaction.

Supplementary Figure 4

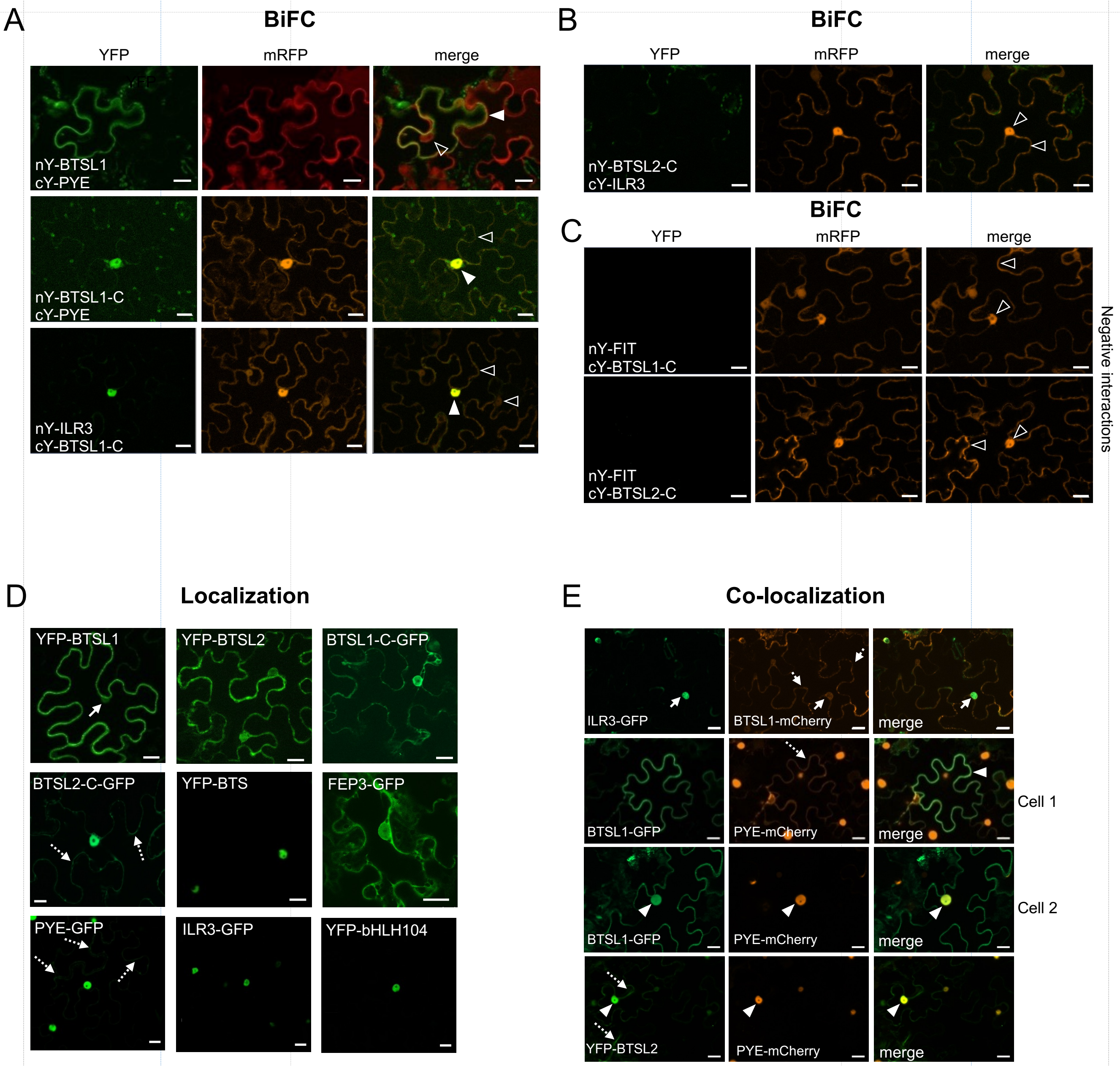

Supplementary Figure 4: Bimolecular fluorescence complementation (BiFC) and localization/co-localization

A, B, BiFC experiments showing protein interactions by YFP fluorescence upon interaction of nY, nYFP- and cY, cYFP-tagged proteins, as indicated. mRFP served as positive transformation control; merge, overlay of YFP and mRFP signals. Generally, only few interactions could be demonstrated using BTSL1/BTSL1-C or BTSL2/BTSL2-C proteins, presumably because of interfering plant factors and low protein stability in plant cells.

A, BTSL1 and BTSL1-C interactions with bHLH proteins. For nY-BTSL1/cY-PYE, positive YFP signals in two independent experiments with 2 infiltrated leaves of one plant, a few BiFC-positive cells per plant. For nY-BTSL1-C/cY-PYE, positive YFP signals in four independent experiments with two plants each, four to five BiFC-positive cells for each infiltrated leaf. For cY-BTSL1-C/nY-ILR3, positive signals in two independent experiments with two plants, five to ten BiFC-positive cells for each infiltrated leaf.

B, BTSL2-C interaction with ILR3.

C, FIT interaction with BTSL1-C and BTSL2-C.

D, Subcellular localization of YFP- and GFP-tagged proteins. Respective protein fusions are indicated. For BTSL1 see confirmation of localization near the plasma membrane for BTSL1-GFP (before and after plasmolysis) and BTSL1-mCherry (Supplemental Figure S5). Non-dashed arrows indicate nucleus, dashed arrow indicates cytoplasm.

E, Subcellular co-localization of GFP/YFP and mCherry-tagged proteins. Arrowheads indicate co-localization (filled arrowheads) and areas of no co-localization (un-filled arrowheads).

A-E, Images were obtained after transient tobacco leaf transformation. Scale bars: 20 µm.

Supplementary Figure 5

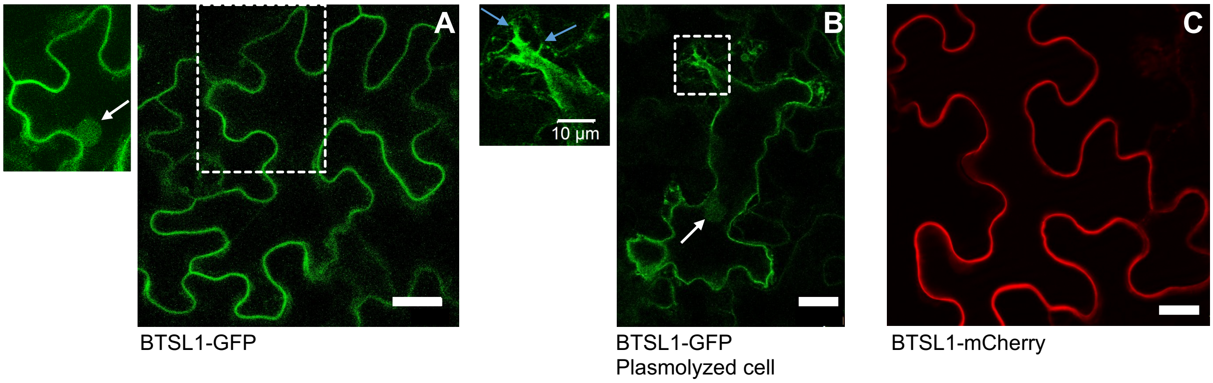

Supplementary Figure 5. Subcellular localization of BTSL1-GFP and BTSL1-mCherry (additional images to Figure 3D, YFP-BTSL1).

Respective protein fusions are indicated. For BTSL1-GFP shown left, before and right, after plasmolysis (Plasmolyzed cell); arrows indicate nucleus. Magnification of the images in A, B show a different layer of the Z stack to better show the nucleus (in A, arrows indicate nucleus) and Hechtian strands (in B, blue arrows indicate Hechtian strands, white arrow indicates nucleus). A, B, imaged by laser-scanning confocal fluorescence microscopy. C, imaged with an epifluorescence microscope and an ApoTome for enhanced resolution. Scale bars: 20 µm.

Supplementary Figure 6

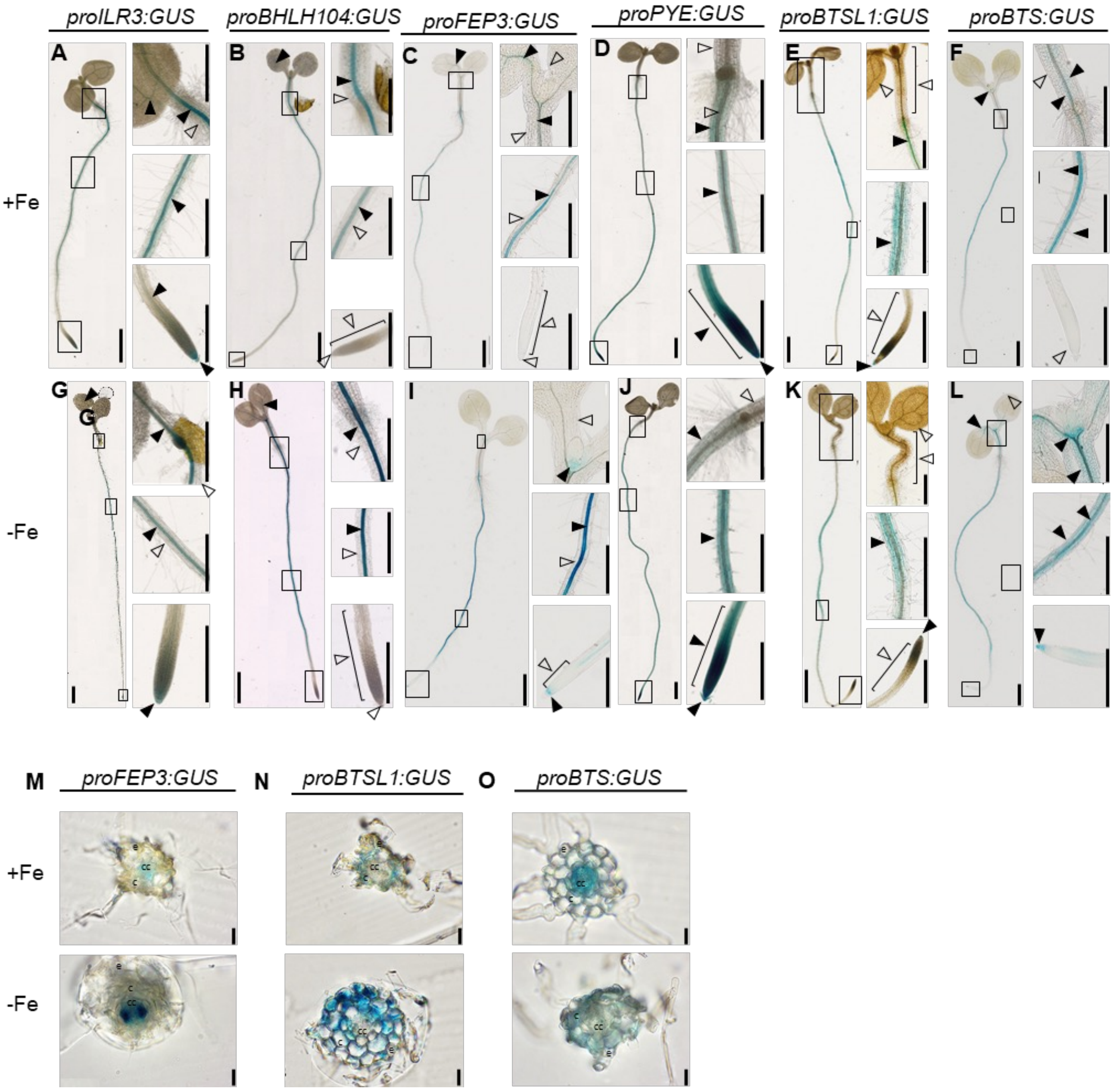

Supplementary Figure 6. Promoter-driven GUS activities of genes encoding the "BTSL-bHLH-FEP3/IMA1 interactome" in roots

GUS reporter activity, driven by promoters of *ILR3*, *BHLH104*, *FEP3*, *PYE*, *BTSL1*, *BTS* promoters. Transgenic Arabidopsis plants with proILR3:GUS (A, G), proBHLH104:GUS (B, H), proFEP3:GUS (C, I), proPYE:GUS (D, J), proBTSL1:GUS (E, K), proBTS:GUS (F, L) in WT background were grown side-by-side in the 6 d system under sufficient (+Fe, A-F) and deficient (-Fe, G-L) Fe supply. Reporter gene expression was visualized with x-gluc resulting in blue staining of the respective tissues. Plants were imaged with brightfield microscopy. Rectangles in whole-seedling images indicate positions of the enlarged image portions. Filled arrowheads indicate selected areas of GUS activity, un-filled arrowheads indicate selected areas without GUS activity. Vascular tissue is indicated by the arrowhead pointing directly at it, non-vascular tissue is indicated by the arrowhead pointing more to an outside area. Scale bars of whole seedling images: 1 mm; of magnifications: 0.5 mm. Cross sections of proFEP3:GUS (M), proBTSL1:GUS (N) and proBTS:GUS (O). Scale bars: 20 µm.

Supplementary Figure 7

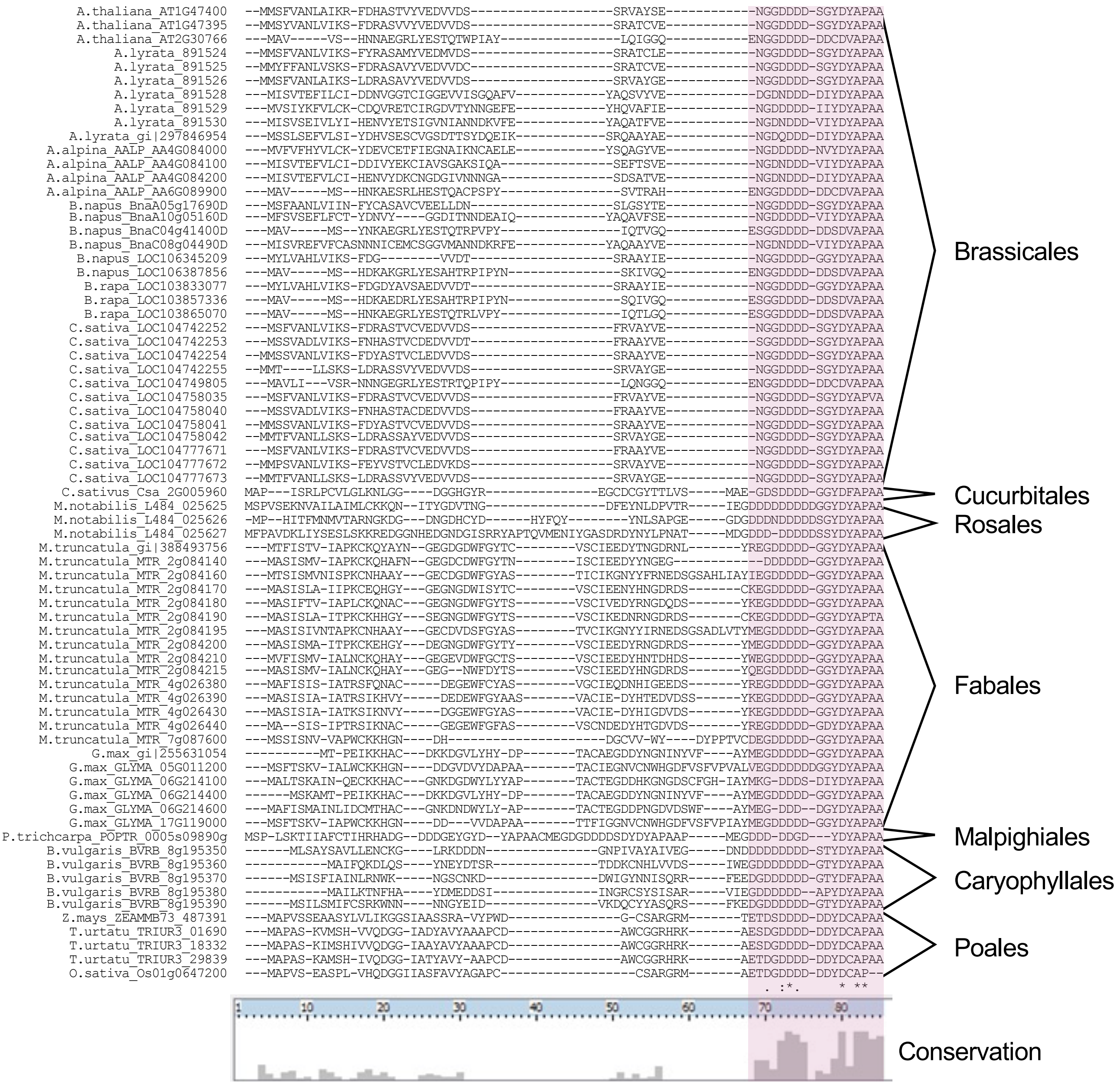

Supplementary Figure 7. FEP3 conserved domain.

Multiple sequence alignment of FEP3/IMA1 (AT1G47400) protein sequence and BLAST search hits of selected angiosperm species, organized by their orders. Corresponds to Figure 6.

Supplementary Figure 8

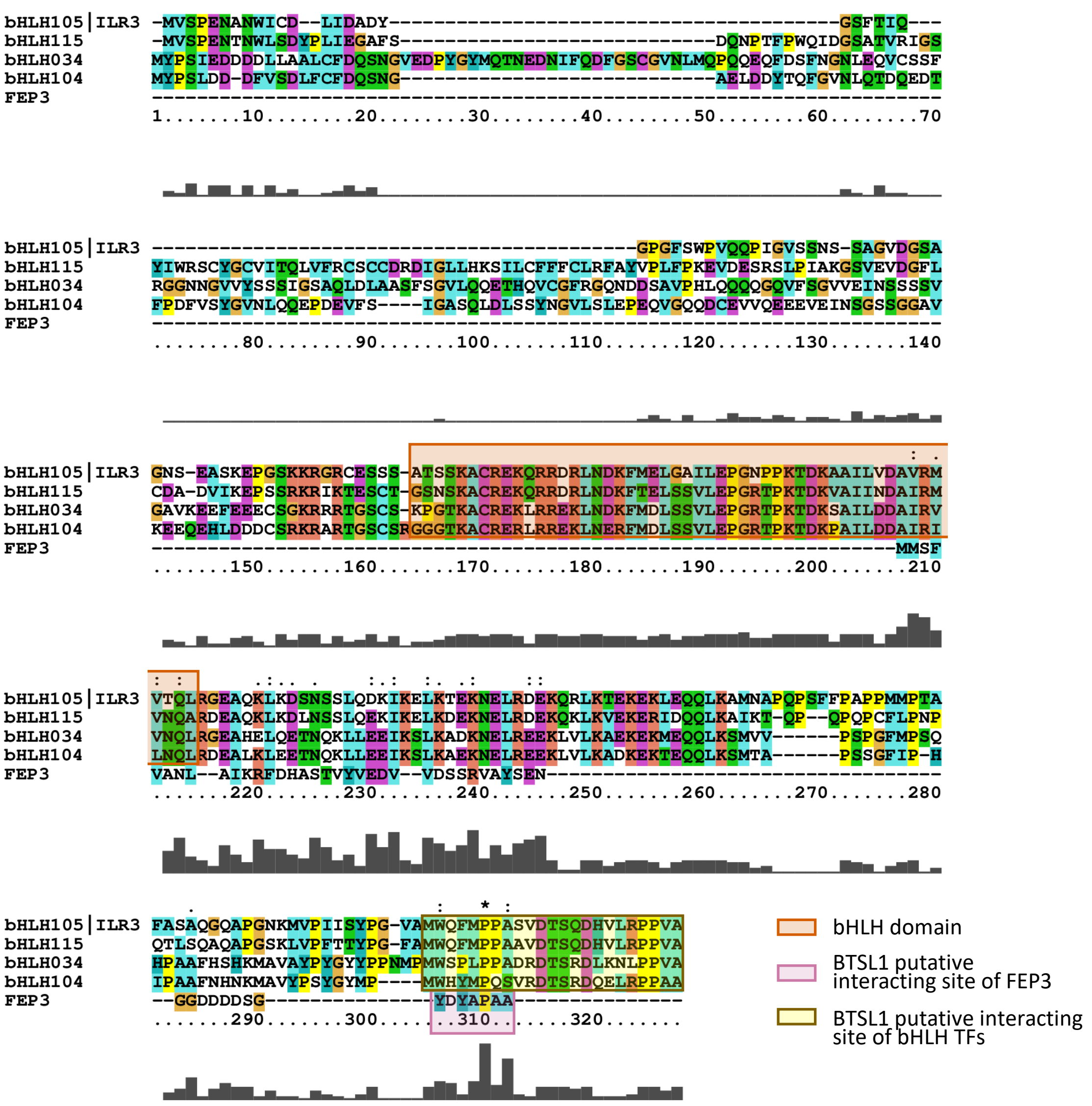

Supplementary Figure 8. Multiple sequence alignment of bHLH IVc TFs and FEP3/IMA1 to identify sequence similarities.

Multiple sequence alignment of ILR3, bHLH115, bHLH034, bHLH104 and FEP3/IMA1 protein sequences. Boxed regions are bHLH domain (orange), the seven C-terminal amino acids of FEP3/IMA1 needed for protein-protein interaction with BTSL1 (see Figure 6) (pink) and the three terminal PVY/PAA amino acids of bHLH factors that may compete with FEP3/IMA1 for binding to BTSL1. The FEP3 sequence YDYAPAA, that may compete for binding to BTSL1, also aligns with the C-terminal end of the bHLH factors. bHLH domain annotation based on information deposited on UniProt (www.uniprot.org). Below is the consensus plot.

Supplementary Figure 9

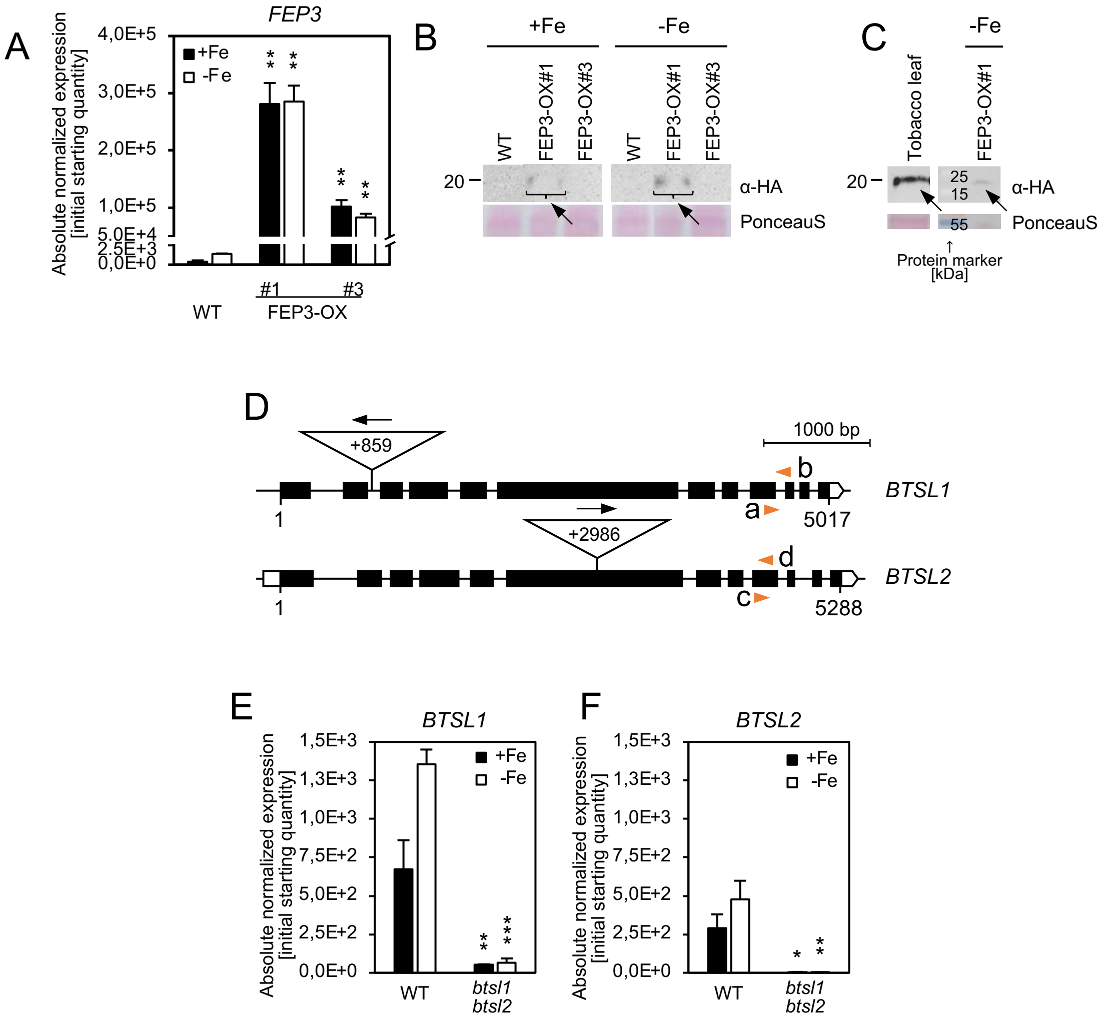

Supplementary Figure 9. Validation of FEP3-OX and *btsl1 btsl2* mutant lines.

A, *FEP3* gene expression in seedlings of WT and two FEP3-OX lines (#1, #3, WT background) generated in this study (see also Supplemental Figure S9D). Plants were grown in the 6 d system with sufficient (+Fe, black bars) or deficient (-Fe, white bars) Fe supply. B, C, Anti-HA immunodetection of HA_3-FEP3 fusion protein (indicated by arrows) in B, -Fe-grown 6-day seedlings of FEP3-OX (#1, #3), B, in tobacco leaves transiently transformed with the HA_3-FEP3 construct, served as positive control, WT served as negative control. Loading control, Ponceau S staining of the membrane (in pink or black). Molecular weight of the protein (in kDa) is indicated. D, Schematic representation of *BTSL1* and *BTSL2* genes and mutant alleles of the *btsl1 btsl2* line. Black boxes indicate exons, white boxes indicate 5' and 3' untranslated regions. Positions of T-DNA insertions (triangles) and primers used for RT-qPCR (orange arrowheads; a, b, c, d) are indicated. Black arrows indicate orientation of left border primers used for genotyping. E, F, Gene expression of E, *BTSL1* and F, *BTSL2* in WT and *btsl1 btsl2* mutant roots. Two-week-old plants were exposed to +Fe or -Fe supply. A, E, F, Data are represented as mean and standard deviation. Asterisks indicate statistically significant differences compared with the respective WT sample of the same growth condition (Student's T-Test, *p<0.05, **p<0.01, ***p<0.001).

Supplementary Figure 10

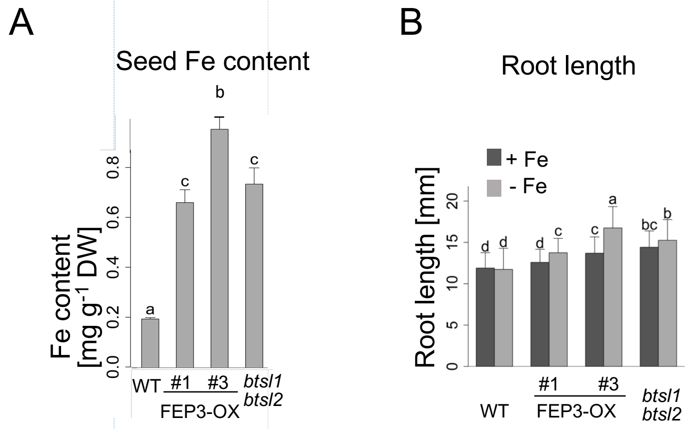

Supplementary Figure 10: Physiological Fe deficiency response phenotypes of FEP3/IMA1 overexpression lines FEP3-Ox and *btsl1 btsl2* mutants

Wild type (WT), two FEP3/IMA1 overexpression lines, FEP3-OX #1 and #3 and *btsl1 btsl2* mutants were analyzed. Further information on the lines is provided in Supplemental Figure S9. Plants were exposed to + and –Fe supply, as indicated. A, Seed Fe content per dry weight (DW), seeds from soil-grown plants (n=3). B, Root lengths of seedlings grown in the 6 d system with +Fe or –Fe, n = 12-20.

Data are represented as mean values with standard deviations. Different letters indicate statistically significant differences (one-way ANOVA and Tukey's post-hoc test, $p<0.05$).

Supplementary Figure 11

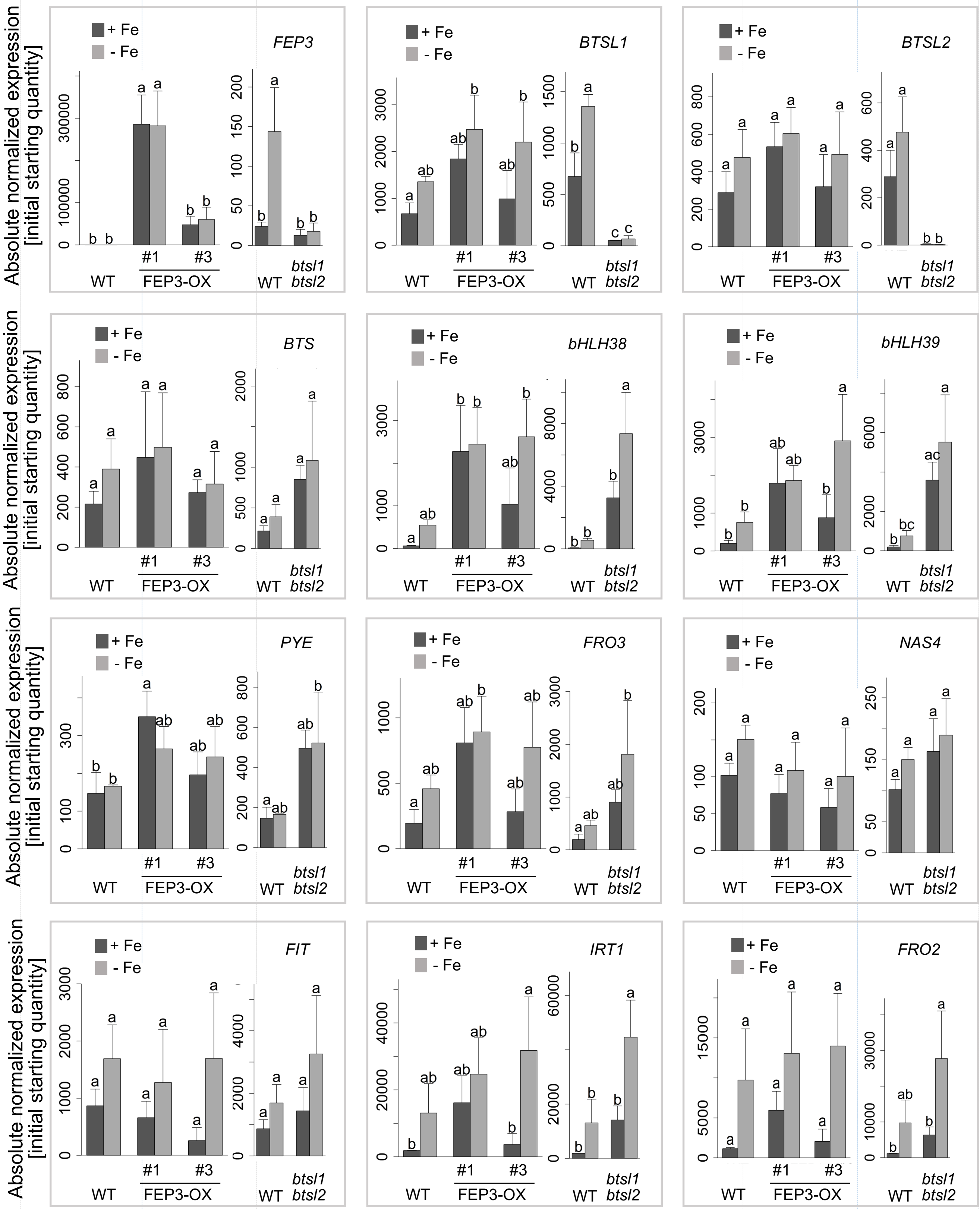

Supplementary Figure 11: Gene expression phenotypes of FEP3/IMA1 overexpression lines FEP3-Ox and *btsl1 btsl2* mutants

Gene expression under +Fe and –Fe in the two-week growth system. Wild type (WT), two FEP3/IMA1 overexpression lines, FEP3-OX #1 and #3 and *btsl1 btsl2* mutants were analyzed. Further information on the lines is provided in Supplemental Figure S9. Analyzed gene expression from upper row, *FEP3*, *BTSL1*, *BTSL2*; middle rows, *BTS*, *BHLH38*, *BHLH39*, *PYE*, *FRO3*, *NAS4*; lower row, *FIT*, *IRT1*, *FRO2*.

Data are represented as mean values with standard deviations. Different letters indicate statistically significant differences (one-way ANOVA and Tukey's post-hoc test, $p<0.05$, $n = 3$).

Supplementary Figure 12

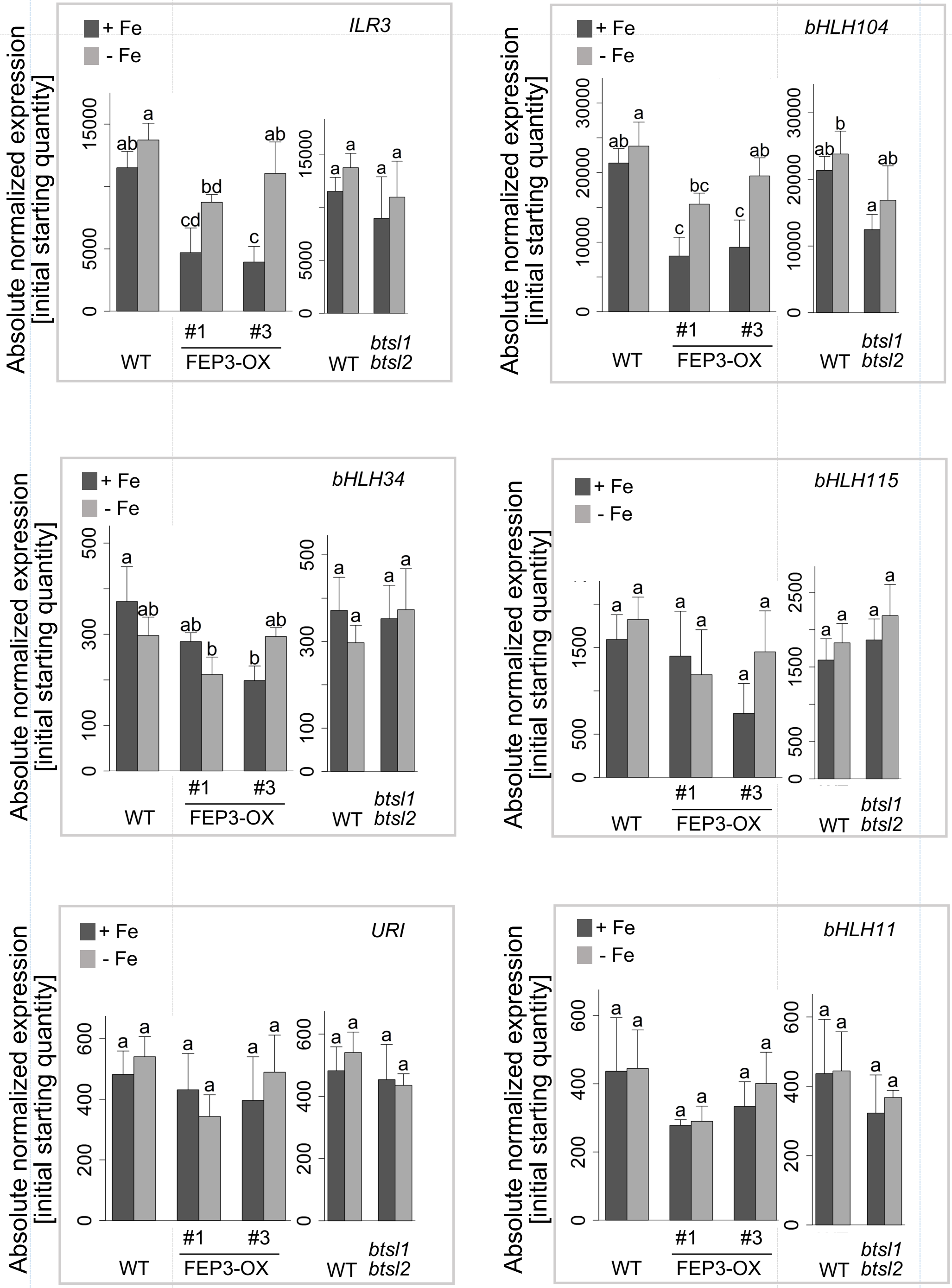

Supplementary Figure 12: *BHLH* subgroup IVc and IVb gene expression phenotypes of FEP3 overexpression lines FEP3-Ox and *btsl1 btsl2* mutants

Gene expression under +Fe and –Fe in the two-week growth system. Wild type (WT), two FEP3/IMA1 overexpression lines, FEP3-OX #1 and #3 and *btsl1 btsl2* mutants were analyzed. Further information on the lines is provided in Supplementary Figure 9. Analyzed gene expression from upper row, *ILR3*, *BHLH104*; middle row, *BHLH034, BHLH115*; lower row, URI/*BHLH121*, *BHLH011*.

Data are represented as mean values with standard deviations. Different letters indicate statistically significant differences (one-way ANOVA and Tukey's post-hoc test, $p<0.05$, n = 3).

Supplementary Figure 13

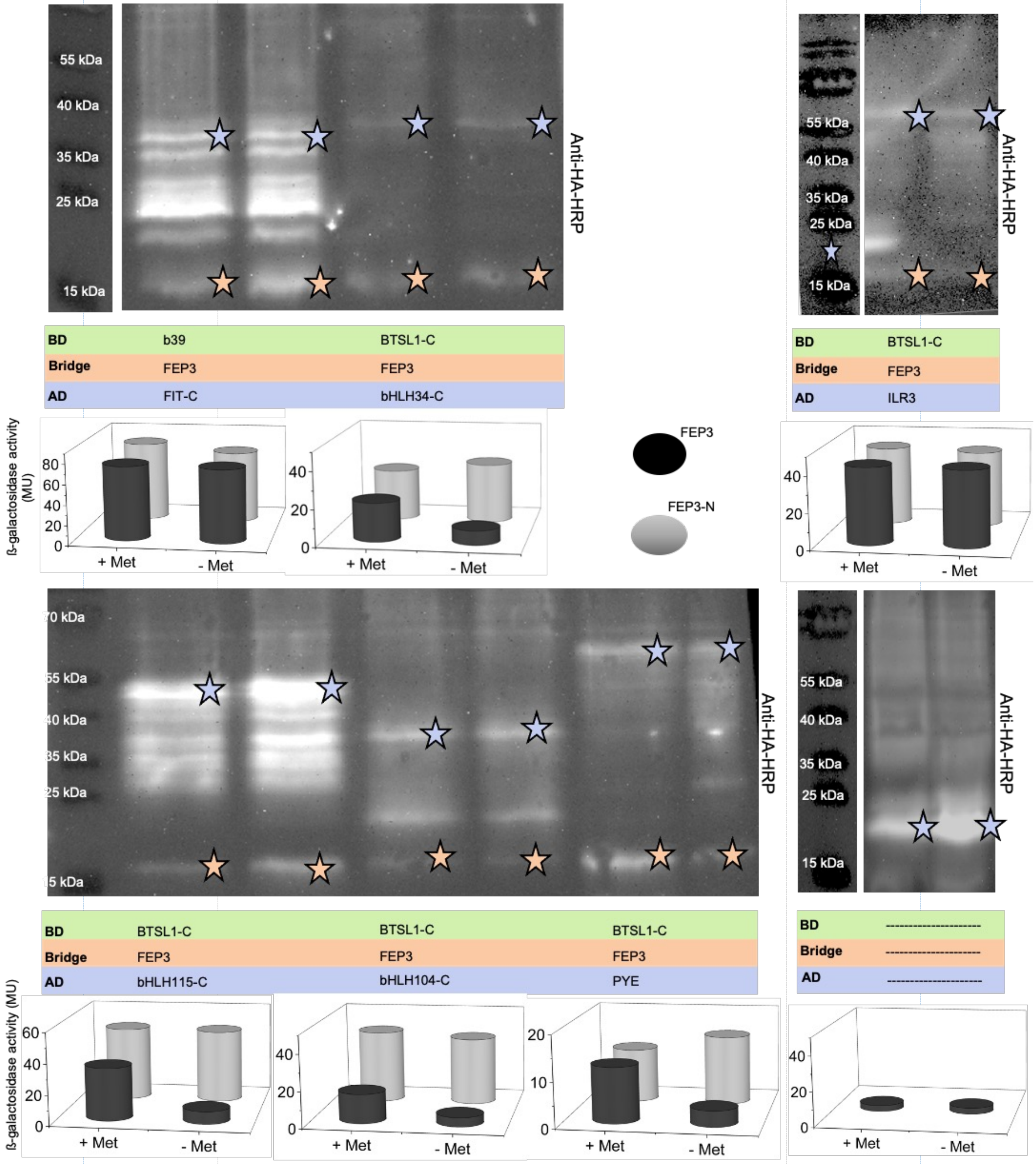

Supplementary Figure 13: FEP3/IMA1 effect on the interaction of BTSL1-C and PYE or bHLH Ivc, quantified by yeast three hybrid (Y3H) assay (additional data)

Yeast cells were grown in SD-LW (+Met, methionine) and SD-LWM (-Met). Upper images, anti-HA immunoblot analyses of HA-tagged proteins, as indicated, to detect orange star, HA-FEP3; blue star, HA-TF; first lane left side, +Met; second lane, right side, -Met. HA-FEP3 is detected at + and -Met. Theoretical molecular mass of HA-tagged proteins: AD-FIT-C (36.66 kDa), AD-bHLH34-C (37.17 kDa), AD-ILR3 (58.28 kDa), AD-bHLH115c (44.8 kDa), AD-bHLH104-C (37.17 kDa), AD-PYE (60.8 kDa, AD (21 kDa), FEP3 (9.9 kDa). Lower image, quantitative analysis of ß-galactosidase activity determined in Miller Units (MU). Quantification of protein interaction strengths of indicated BD/AD protein pairs in the presence of effector protein FEP3/IMA1 and FEP3-N, at +/-Met, respectively.

Supplementary Figure 14

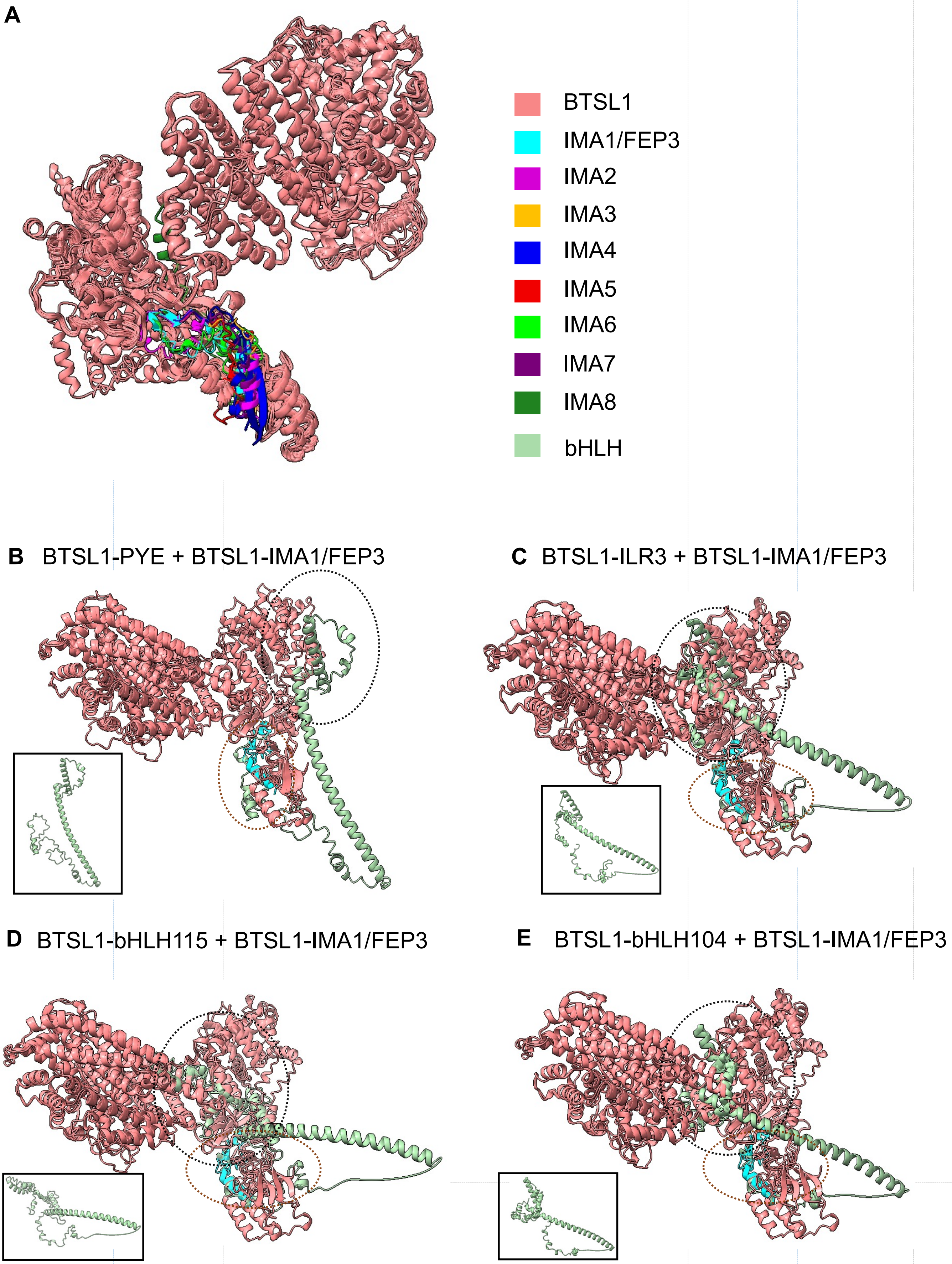

Supplementary Figure 14: Structural alignment of Alphafold-Multimer predicted BTSL1-bHLH-IMA/FEP3 protein structures.

A, Structural alignment of predicted structures of eight BTSL1-IMA proteins, each IMA protein colored differently. All IMA proteins align at a similar region close to the MC site of BTSL1. BTSL1 colored in pink. IMA1-8 colored as in legend.

B,C,D,E, Structural alignment of BTSL1-IMA1/FEP3 with A, BTSL1-PYE, B, BTSL1-ILR3, C, BTSL1-bHLH115, D, BTSL1-bHLH104. BTSL1 colored in pink. IMA1/FEP3 colored in aqua blue. PYE, ILR3, bHLH115 and bHLH104 colored in mint green. Interface A, marked in the dotted black circle, Interface B, marked in the dotted brown circle. bHLH protein structures are represented in inset boxes
