# Supplementary material for: The Iron Deficiency-Regulated Small Protein Effector FEP3/IRON MAN1 Modulates Interaction of BRUTUS-LIKE1 With bHLH Subgroup IVc and POPEYE Transcription Factors
Source: Front Plant Sci. 2022 Jun 10;13:930049. doi: 10.3389/fpls.2022.930049 (PMC9226616; doi:10.3389/fpls.2022.930049)
Supplement: Supplementary file 2 [file Table_1.docx]

Supplementary Table 1. List of candidates tested in Y2H assays in this work.

**AGI Short name Description**

1. 23 Proteins tested in pair-wise Y2H combinations (targeted Y2H screen)

FIT-dependent (cluster 2, 3) ^1^

AT2G2816 FIT FER-LIKE Fe DEFICIENCY INDUCED TRANSCRIPTION

FACTOR, bHLH29

AT1G34760 GRF11 GENERAL REGULATORY FACTOR 11, 14-3-3 protein

AT3G06890 UP1 unknown protein (128 aa)

AT3G07720 KELCH galactose oxidase/kelch repeat superfamily protein

AT3G12900 S8H SCOPOLETIN 8- HYDROXYLASE

AT5G56080 NAS2 NICOTIANAMINE SYNTASE2

FIT-independent (cluster 1) ^1^

AT1G12030 DUF506 DOMAIN OF UNKNOWN FUNCTION506

AT1G47400 FEP3/IMA1 FE-UPTAKE-INDUCING PEPTIDE3, IRONMAN1

AT1G48300 DGAT3 DIACYLGLYCEROL ACYLTRANSFERASE3, 2Fe-2S cluster

AT1G56430 NAS4 NICOTIANAMINE SYNTHASE4

AT1G74770 BTSL1 BRUTUS-LIKE1, RING E3 ligase

AT3G18290 BTS BRUTUS, RING E3 ligase

AT3G47640 PYE POPEYE, bHLH TF

AT3G56360 UP2 unknown protein

AT3G56980 bHLH39 bHLH TF

AT5G05250 UP3 unknown protein

AT5G53450 ORG1 OBP3-RESPONSIVE GENE1, predicted protein kinase activity

AT5G48850 SDI1 SULPHUR DEFICIENCY-INDUCED1

Other not co-expressed TFs regulating Fe deficiency responses and other not co-expressed candidates*

AT1G32380 PRS2 PHOSPHORIBOSYL PYROPHOSPHATE SYNTHASE2

AT1G52120 JAL12 PHOSPHORIBOSYL PYROPHOSPHATE SYNTHASE2

AT1G56160 MYB72 MYB TF

AT4G14410 bHLH104 bHLH TF

AT5G54680 ILR3 IAA-LEUCINE RESISTANT3, bHLH105, bHLH TF

1. Additional Fe-response-related proteins used for validation and extension

AT4G36060 bHLH11 bHLH TF

AT3G23210 bHLH34 bHLH TF

AT1G51070 bHLH115 bHLH TF

AT3G19860 bHLH121 URI, bHLH TF

AT1G18910 BTSL2 BRUTUS-LIKE1, RING E3 ligase

^1^Schwarz and Bauer, 2020; *not co-expressed with the other Fe deficiency response genes. Short name and description according to The Arabidopsis Information Resource (TAIR), 10.0 genome release, if no short name available, we provided a shortened description version.
