# Supplementary material for: The Iron Deficiency-Regulated Small Protein Effector FEP3/IRON MAN1 Modulates Interaction of BRUTUS-LIKE1 With bHLH Subgroup IVc and POPEYE Transcription Factors
Source: Front Plant Sci. 2022 Jun 10;13:930049. doi: 10.3389/fpls.2022.930049 (PMC9226616; doi:10.3389/fpls.2022.930049)
Supplement: Supplementary file 3 [file Table_2.docx]

****Supplementary**** Table 2. Primers used in this study.

fw = forward, rev = reverse, s = stop, ns = no stop, d = delta a.k.a. deletion, -C = C-term, -CC = 25 C-terminal aa, -N = N-term, (att)B1/B2/B3/B4 = Gateway B attachment sites for BP reaction.

| **Primer name** | **Sequence 5‘** $\boldsymbol{\to}$ **3‘** | **Purpose** | **Origin** |
| --- | --- | --- | --- |
| **Amplification of full-length CDS or deletion constructs for Y2H, BiFC, (co-) localization and OX lines** | | | |
| BTSL1_B1 fw | GGGGACAAGTTTGTACAAAAAAGCAGGCTTCATGGGAGGCGGAAATCTTC | cloning of BTSL1, BTSL1-N, BTSL1-dRH, BTSL1-6G (attB1) | This study |
| BTSL1s_B2 rev | GGGGACCACTTTGTACAAGAAAGCTGGGTCCTAAAGGAGCCTTGAGTTGTAG | cloning of BTSL1, BTSL1-C, BTSL1-C.4, BTSL1-dRH, BTSL1-6G (attB2) | This study |
| BTSL1ns_B2 rev | GGGGACCACTTTGTACAAGAAAGCTGGGTCAAGGAGCCTTGAGTTGTAGGA | cloning of BTSL1, BTSL1-C, no stop codon (attB2) | This study |
| BTSL1-Ns_B2 rev | GGGGACCACTTTGTACAAGAAAGCTGGGTCCTAAGGAGGCTGATTCATAATACCTG | cloning of BTSL1-N (attB2) | This study |
| BTSL1-C_B1 fw | GGGGACAAGTTTGTACAAAAAAGCAGGCTTCATGCCTAATTACAAGGTTGAAGTTGGC | cloning of BTSL1-C, BTSL1-C.1, BTSL1-C.2 (attB1) | This study |
| BTSL1_B3 fw | GGGGACAACTTTGTATAATAAAGTTGTAATGGGAGGCGGAAATCTTC | cloning of BTSL1 (attB3) | This study |
| BTSL1s_B4 rev | GGGGACAACTTTGTATAGAAAAGTTGGGTGCTAAAGGAGCCTTGAGTTGTAG | cloning of BTSL1-C (attB4) | This study |
| BTSL1-C.1s_B2 rev | GGGGACCACTTTGTACAAGAAAGCTGGGTCCTAGTTGCAGTAAGGACAGTGATAA | cloning of BTSL1-C.1 (attB2) | This study |
| BTSL1-C.2s_B2 rev | GGGGACCACTTTGTACAAGAAAGCTGGGTCCTAGCACTTTTCCCTGCAAACAT | cloning of BTSL1-C.2 (attB2) | This study |
| BTSL1-C.3_B1 fw | GGGGACAAGTTTGTACAAAAAAGCAGGCTTCATGGAAGAAGCTGATCACTCGGT | cloning of BTSL1-C.3 (attB1) | This study |
| BTSL1-C.3s_B2 rev | GGGGACCACTTTGTACAAGAAAGCTGGGTCCTAATCAGGCATCTTCTCTTCTGC | cloning of BTSL1-C.3 (attB2) | This study |
| BTSL1-C.4_B1 fw | GGGGACAAGTTTGTACAAAAAAGCAGGCTTCATGTCACGTACCTTAGTAGAGC | cloning of BTSL1-C.4 (attB1) | This study |
| BTSL1_dRH rev | CTTTTCCCTGCAAACTGACATACAAGCATTGCAT | cloning of BTSL1-dRH for overlap extension PCR (N-term part) | This study |
| BTSL1_dRH fw | AATGCTTGTATGTCAGTTTGCAGGGAAAAGTG | Cloning of BTSL1-dRH for overlap extension PCR (C-term part) | This study |
| BTSL1_dRH-G rev | GCAAACGCCTCCGCCTCCGCCTCCTGACATACAAGCATTGCAT | cloning of BTSL1-6G for overlap extension PCR (C-term part) | This study |
| BTSL1_dRH-G fw | ATGTCAGGAGGCGGAGGCGGAGGCGTTTGCAGGGAAAAGTG | Cloning of BTSL1-6G for overlap extension PCR (C-term part) | This study |
| BTSL1_dMC fw | AAATGCAATGCTTGTTTAGAAGATAATTGTCCGATTTGCC | Cloning of BTSL1-dMC for overlap extension | This study |
| BTSL1_dMC rev | ACAATTATCTTCTAAACAAGCATTGCATTTCATGC | Cloning of BTSL1-dMC for overlap extension | This study |
| BTSL2_B1 fw | GGGGACAAGTTTGTACAAAAAAGCAGGCTTCATGGGAGTCGGAGATCCT | cloning of BTSL2 (attB1) | This study |
| BTSL2s_B2 rev | GGGGACCACTTTGTACAAGAAAGCTGGGTCCTAGAAAAGTCTGGTGTTGTAGG | cloning of BTSL2 , BTSL2-C (attB2) | This study |
| BTSL2-C_B3 fw | GGGGACAACTTTGTATAATAAAGTTGTAATGGGAGTCGGAGATCCT | cloning of BTSL2-C (attB3) | This study |
| BTS_B1 fw | GGGGACAAGTTTGTACAAAAAAGCAGGCTTCATGGCGACGCCGTTACCA | cloning of BTS (attB1) | This study |
| BTSs_B2 rev | GGGGACCACTTTGTACAAGAAAGCTGGGTCTCAGGATGAGGTTGAGCAGT | cloning of BTS (attB2) | This study |
| BTSs_B4 rev | GGGGACAACTTTGTATAGAAAAGTTGGGTGTCAGGATGAGGTTGAGCAGT | cloning of BTS (attB4) | This study |
| FEP3_B1 fw | GGGGACAAGTTTGTACAAAAAAGCAGGCTTCATGATGTCTTTTGTCGCAAAC | cloning of FEP3, FEP3-N, FEP3-d7 (attB1) | This study |
| FEP3s_B2 rev | GGGGACCACTTTGTACAAGAAAGCTGGGTCTCACGCAGCAGGAGCATA | cloning of FEP3, FEP3-C (attB2) | This study |
| FEP3ns_B2 rev | GGGGACCACTTTGTACAAGAAAGCTGGGTCCGCAGCAGGAGCATAATC | cloning of FEP3, proFEP3-FEP3, no stop codon (attB2) | This study |
| FEP3-Ns_B2 rev | GGGGACCACTTTGTACAAGAAAGCTGGGTCTCATACCACATCTTCAACATACACG | cloning of FEP3-N (attB2) | This study |
| FEP3-C_B1 fw | GGGGACAAGTTTGTACAAAAAAGCAGGCTTCATGGATAGTTCTCGAGTGGCATATAG | cloning of FEP3-C (attB1) | This study |
| FEP3-d7s_B2 rev | GGGGACCACTTTGTACAAGAAAGCTGGGTCTCATCAGCCACTGTCATCGTC | cloning of FEP3-d7 (attB2) | This study |
| ILR3_B1 fw | GGGGACAAGTTTGTACAAAAAAGCAGGCTTCATGGTGTCACCCGAAAACG | cloning of ILR3, ILR3-d25 (attB1) | This study |
| ILR3s_B2 rev | GGGGACCACTTTGTACAAGAAAGCTGGGTCTTAAGCAACAGGAGGACGAAG | cloning of ILR3 (attB2) | This study |
| ILR3ns_B2 rev | GGGGACCACTTTGTACAAGAAAGCTGGGTCAGCAACAGGAGGACGAAG | cloning of ILR3, no stop codon (attB2) | This study |
| ILR3_B3 fw | GGGGACAACTTTGTATAATAAAGTTGTAATGGTGTCACCCGAAAACG | cloning of ILR3 (attB3) | This study |
| ILR3s_B4 rev | GGGGACAACTTTGTATAGAAAAGTTGGGTGTTAAGCAACAGGAGGACGAAG | cloning of ILR3 (attB4) | This study |
| ILR3-d25s_B2 rev | GGGGACCACTTTGTACAAGAAAGCTGGGTCTTATCCTGGGTAACTGATGAT | cloning of ILR3-d25 (attB2) | This study |
| ILR3-CC_B1 fw | GGGGACAAGTTTGTACAAAAAAGCAGGCTTCATGGTTGCCATGTGG | cloning of ILR3-CC (attB1) | This study |
| bHLH104_B1 fw | GGGGACAAGTTTGTACAAAAAAGCAGGCTTCATGTATCCTTCTCTCGACGA | cloning of bHLH104 (attB1) | This study |
| bHLH104s_B2 rev | GGGGACCACTTTGTACAAGAAAGCTGGGTCTTAAGCAGCAGGAGGCCTG | cloning of bHLH104, bHLH104-C, bHLH104-CC (attB2) | This study |
| bHLH104-C_B1 fw | GGGGACAAGTTTGTACAAAAAAGCAGGCTTCATGGCTCT | cloning of bHLH104-C, bHLH104-C-d25 (attB1) | This study |
| bHLH104-d25s_B2 rev | GGGGACCACTTTGTACAAGAAAGCTGGGTCTTAGTAACCGTAACTTGGATAAAC | cloning of bHLH104-C-d25 (attB2) | This study |
| bHLH104-CC_B1 fw | GGGGACAAGTTTGTACAAAAAAGCAGGCTTCATGATGCCAATGTGGC | cloning of bHLH104-CC (attB1) | This study |
| PYE_B1 fw | GGGGACAAGTTTGTACAAAAAAGCAGGCTTCATGGTATCGAAAACTCCTTC | cloning of PYE (attB1) | This study |
| PYEs_B2 rev | GGGGACCACTTTGTACAAGAAAGCTGGGTCTCATTCACTGGCTTTCAGCC | cloning of PYE (attB2) | This study |
| PYEns B2 rev | GGGGACCACTTTGTACAAGAAAGCTGGGTCTTCACTGGCTTTCAGCC | cloning of PYE, no stop codon (attB2) | This study |
| PYE_B3 fw | GGGGACAACTTTGTATAATAAAGTTGTAATGGTATCGAAAACTCCTTC | cloning of PYE (attB3) | This study |
| PYEs_B4 rev | GGGGACAACTTTGTATAGAAAAGTTGGGTGTCATTCACTGGCTTTCAGCC | cloning of PYE (attB4) | This study |
| FIT_B1 fw | GGGGACAAGTTTGTACAAAAAAGCAGGCTTCATGGAAGGAAGAGTCAACG | cloning of FIT (attB1) | This study |
| FITs_B2 rev | GGGGACCACTTTGTACAAGAAAGCTGGGTCTCAAGTAAATGACTTGATG | cloning of FIT, FIT-C (attB2) | This study |
| FIT-C_B1 rev | GGGGACAAGTTTGTACAAAAAAGCAGGCTTAACTCAACCTTTTCGCGGTATC | cloning of FIT-C (attB1) | (Gratz et al., 2019) |
| FIT_B3 fw | GGGGACAACTTTGTATAATAAAGTTGTAATGGAAGGAAGAGTCAACG | cloning of FIT (attB3) | This study |
| bHLH38_B1 fw | GGGGACAAGTTTGTACAAAAAAGCAGGCTTCATGTGTGCATTAGTCCCTTCATTT | cloning of bHLH38 (attB1) | This study |
| bHLH38_B2 rev | GGGGACCACTTTGTACAAGAAAGCTGGGTCCTAGTTAAACGAGTTTTCACATT | cloning of bHLH38 (attB2) | This study |
| bHLH39_B1 fw | GGGGACAAGTTTGTACAAAAAAGCAGGCTTCATGTGTGCATTAGTACCTC | cloning of bHLH39 (attB1) | This study |
| bHLH39_B2 rev | GGGGACCACTTTGTACAAGAAAGCTGGGTCTCATATATATGAGTTTCCAC | cloning of bHLH39 (attB2) | This study |
| bHLH100_B1 fw | GGGGACAAGTTTGTACAAAAAAGCAGGCTTCATGTGTGCACTTGTCCCTCCATT | cloning of bHLH100 (attB1) | This study |
| bHLH100_B2 rev | GGGGACCACTTTGTACAAGAAAGCTGGGTCTCATGTAAACGAGTGTCCACATT | cloning of bHLH100 (attB2) | This study |
| bHLH101_B1 fw | GGGGACAAGTTTGTACAAAAAAGCAGGCTTCATGGAGTATCCATGGCTGCAGTC | cloning of bHLH101 (attB1) | This study |
| bHLH101_B2 rev | GGGGACCACTTTGTACAAGAAAGCTGGGTCTTATGATTGGCGTAATCCCAAGA | cloning of bHLH101 (attB2) | This study |
| MYB72_B1 fw | GGGGACAAGTTTGTACAAAAAAGCAGGCTTCATGGGGAAAGGAAGAGCAC | cloning of MYB72 (attB1) | This study |
| MYB72_B2 rev | GGGGACCACTTTGTACAAGAAAGCTGGGTCTCATAGACATACTTCTCCGA | cloning of MYB72 (attB2) | This study |
| DGAT3_B1 fw | GGGGACAAGTTTGTACAAAAAAGCAGGCTTCATGGAGAAGGAGAAGAAGGC | cloning of DGAT3 (attB1) | This study |
| DGAT3s_B2 rev | GGGGACCACTTTGTACAAGAAAGCTGGGTCTCAATATGAGACAGAACCGAGT | cloning of DGAT3 (attB2) | This study |
| DUF506_B1 fw | GGGGACAAGTTTGTACAAAAAAGCAGGCTTCATGGTAGAGATAGGAGGACGA | cloning of DUF506 (attB1) | This study |
| DUF506s_B2 rev | GGGGACCACTTTGTACAAGAAAGCTGGGTCCTAAAATATTTGCAACCCCACTT | cloning of DUF506 (attB2) | This study |
| GRF11_B1 fw | GGGGACAAGTTTGTACAAAAAAGCAGGCTTCATGGAGAACGAGAGAGCG | cloning of GRF11 (attB1) | This study |
| GRF11s_B2 rev | GGGGACCACTTTGTACAAGAAAGCTGGGTCTTAGATTTTGTTTACCTCATCTTG | cloning of GRF11 (attB2) | This study |
| JAL12_B1 fw | GGGGACAAGTTTGTACAAAAAAGCAGGCTTCATGTCTCAAGATTCGAATGC | cloning of JAL12 (attB1) | This study |
| JAL12s_B2 rev | GGGGACCACTTTGTACAAGAAAGCTGGGTCTCACCAGCTTGTGTGTATG | cloning of JAL12 (attB2) | This study |
| KELCH_B1 fw | GGGGACAAGTTTGTACAAAAAAGCAGGCTTCATGGCAGCGACTCCAATG | cloning of KELCH (attB1) | This study |
| KELCHs_B2 rev | GGGGACCACTTTGTACAAGAAAGCTGGGTCTCACTTGAGTAGAGTGTTGG | cloning of KELCH (attB2) | This study |
| NAS2_B1 fw | GGGGACAAGTTTGTACAAAAAAGCAGGCTTCATGGCTTGCGAAAACAACCT | cloning of NAS2 (attB1) | This study |
| NAS2s_B2 rev | GGGGACCACTTTGTACAAGAAAGCTGGGTCTTACTCGATGGCACTATACTCCT | cloning of NAS2 (attB2) | This study |
| NAS4_B1 fw | GGGGACAAGTTTGTACAAAAAAGCAGGCTTCATGGGTTATTGCCAAGACGA | cloning of NAS4 (attB1) | This study |
| NAS4s_B2 rev | GGGGACCACTTTGTACAAGAAAGCTGGGTCCTAGGTAAGTTGTTCTTCATTAGCA | cloning of NAS4 (attB2) | This study |
| ORG1_B1 fw | GGGGACAAGTTTGTACAAAAAAGCAGGCTTCATGGCACTTTGTGGTGTTTG | cloning of ORG1 (attB1) | This study |
| ORG1s_B2 rev | GGGGACCACTTTGTACAAGAAAGCTGGGTCCTACATAGACTTATGGTCCAAGC | cloning of ORG1 (attB2) | This study |
| PRS2_B1 fw | GGGGACAAGTTTGTACAAAAAAGCAGGCTTCATGGCGTCGTTGGCTCT | cloning of PRS2 (attB1) | This study |
| PRS2s_B2 rev | GGGGACCACTTTGTACAAGAAAGCTGGGTCTCAAAGGAAAATACTACTAACGGAG | cloning of PRS2 (attB2) | This study |
| S8H_B1 fw | GGGGACAAGTTTGTACAAAAAAGCAGGCTTCATGGGTATCAATTTCGAGGAC | cloning of S8H (attB1) | This study |
| S8Hs_B2 rev | GGGGACCACTTTGTACAAGAAAGCTGGGTCTCACTCGGCACGTGC | cloning of S8H (attB2) | This study |
| SDI1_B1 fw | GGGGACAAGTTTGTACAAAAAAGCAGGCTTCATGGAGAGAAGCTTGAAGAA | cloning of SDI1 (attB1) | This study |
| SDI1s_B2 rev | GGGGACCACTTTGTACAAGAAAGCTGGGTCCTAGCAAACTAATGTATTTCTAAAAG | cloning of SDI1 (attB2) | This study |
| UIP1_B1 fw | GGGGACAAGTTTGTACAAAAAAGCAGGCTTCATGTATCAAGATCGTCAAGGC | cloning of UP1 (attB1) | This study |
| UIP1s_B2 rev | GGGGACCACTTTGTACAAGAAAGCTGGGTCTCAGTCATCGGAACCATCA | cloning of UP1 (attB2) | This study |
| UIP2_B1 fw | GGGGACAAGTTTGTACAAAAAAGCAGGCTTCATGGCGACGTCTGCGA | cloning of UP2 (attB1) | This study |
| UIP2s_B2 rev | GGGGACCACTTTGTACAAGAAAGCTGGGTCTCATTGACCAGTCTGCACC | cloning of UP2 (attB2) | This study |
| UIP3_B1 fw | GGGGACAAGTTTGTACAAAAAAGCAGGCTTCATGGCGTATGCAAAGATCG | cloning of UP3 (attB1) | This study |
| UIP3s_B2 rev | GGGGACCACTTTGTACAAGAAAGCTGGGTCTCAATTTCCCACAAGCCAAC | cloning of UP3 (attB2) | This study |
| bHLH11_B1 fw | GGGGACAAGTTTGTACAAAAAAGCAGGCTTCATGGATCAACCAATGAAAC | Cloning of bHLH11 (attB1) | This study |
| bHLH11_B2 rev | GGGGACCACTTTGTACAAGAAAGCTGGGTCTTATGGCTTCAACATGTCA | Cloning of bHLH11 (attB2) | This study |
| URI_B1 fw | GGGGACAAGTTTGTACAAAAAAGCAGGCTTCATGGGGATAAGAGAAAATG | Cloning of URI (attB1) | This study |
| URI_B2 rev | GGGGACCACTTTGTACAAGAAAGCTGGGTCTCATTTTGCATCATCAGGTT | Cloning of URI (attB2) | This study |
| bHLH34-C_B1 fw | GGGGACAAGTTTGTACAAAAAAGCAGGCTTCATGAGAGGTGAAGCTC | Cloning of bHLH34-C (attB1) | This study |
| bHLH34-C_B2 rev | GGGGACCACTTTGTACAAGAAAGCTGGGTCTTAAGCAACAGGAGGAA | Cloning of bHLH34-C (attB2) | This study |
| bHLH115_B1 fw | GGGGACAAGTTTGTACAAAAAAGCAGGCTTCATGGTGTCTCCGGAGAA | Cloning of bHLH115 (attB1) | This study |
| bHLH115_B2 rev | GGGGACCACTTTGTACAAGAAAGCTGGGTCTTAAGCAACTGGAGGACG | Cloning of bHLH115, bHLH115-C (attB2) | This study |
| bHLH115-C_B1 fw | GGGGACAAGTTTGTACAAAAAAGCAGGCTTCATGAAAGAACCAAGTTCAAGGA | Cloning of bHLH115-C (attB1) | This study |
| bHLH34_B1 fw | GGGGACAAGTTTGTACAAAAAAGCAGGCTTCATGTATCCATCAATCGAAG | Cloning of bHLH34 (attB1) | This study |
| bHLH34_B2 rev | GGGGACCACTTTGTACAAGAAAGCTGGGTCTTAAGCAACAGGAGGAAG | Cloning of bHLH34 (attB2) | This study |
| **Amplification of promoter regions for GUS lines** | | | |
| proBTSL1_-880_B1 fw | GGGGACAAGTTTGTACAAAAAAGCAGGCTTCAGTATTGATTTTGTGAGCCCAATT | cloning of BTSL1 promoter (attB1) | This study |
| proBTSL1_-880_B2 rev | GGGGACCACTTTGTACAAGAAAGCTGGGTCCCACCGCAACAACAATAACG | cloning of BTSL1 promoter (attB2) | This study |
| proBTS_-2994_B1 fw | GGGGACAAGTTTGTACAAAAAAGCAGGCTTCATGAGATGAAATGTCTTATCTTTAT | cloning of BTS promoter (attB1) | This study |
| proBTS_-2994_B2 rev | GGGGACCACTTTGTACAAGAAAGCTGGGTCTTCCCCCAAAGCTTATCTCCGTTTT | cloning of BTS promoter (attB2) | This study |
| proPYE_-1120_B1 fw | GGGGACAAGTTTGTACAAAAAAGCAGGCTTCACCGCAAAACTATATATAGTATTT | cloning of PYE promoter (attB1) | This study |
| proPYE_-1120_B2 rev | GGGGACCACTTTGTACAAGAAAGCTGGGTCCTTTGCTTTTATTACAGAACAAGA | cloning of PYE promoter (attB2) | This study |
| proFEP3_-1614_B1 fw | GGGGACAAGTTTGTACAAAAAAGCAGGCTTCGGCACAAAGAAATCAGACCAAT | cloning of FEP3 promoter (attB1) | This study |
| proFEP3_-1614_B2rev | GGGGACCACTTTGTACAAGAAAGCTGGGTCTGATATTTTTTGTTGGATATGAATGAAAGT | cloning of FEP3 promoter (attB2) | This study |
| **TAIL PCR and genotyping** | | | |
| S1_AL2_LB | ATGCTCTTACGTTGTTGTCGGG | TAIL, binds in T-DNA LB of pAlligator2 |  |
| S2_AL2_LB | ACCACTCATCATAGCTCCGC | TAIL, binds in T-DNA LB of pAlligator2 |  |
| S3_AL2_LB | TTCAGTACATTAAAAACGTCCGC | TAIL, binds in T-DNA LB of pAlligator2, genotyping of FEP3-OX lines |  |
| AD1 | NGTCGASWGANAWGAA | TAIL | AD2 in (Liu et al., 1995) |
| AD2 | TGWGNAGSANCASAG | TAIL | AD1 in (Liu and Whittier, 1995) |
| AD3 | AGWGNAGWANCAWAGG | TAIL | AD2 in (Liu and Whittier, 1995) |
| AD4 | STTGNTASTNCTNTGC | TAIL | AD2 in (Tsugeki et al., 1996) |
| AD5 | NTCGASTWTSGWGTT | TAIL | AD1 in (Liu et al., 1995) |
| AD6 | WGTGNAGWANCANAGA | TAIL | AD3 in (Liu et al., 1995) |
| LBb1.3 | ATTTTGCCGATTTCGGAAC | *btsl1 btsl2* DM genotyping | Salk Institute Genomic Analysis Laboratory |
| FEP3-OX1_chr5 fw | GCAAACCAAGCTTCCATGC | FEP3-OX#1 genotyping | This study |
| FEP3-OX1_chr5 rev | CTCGCCTCCAATACCTCCTT | FEP3-OX#1 genotyping | This study |
| FEP3-OX3_chr1 fw | CGAATGTACTTCGCTGACTTT | FEP3-OX#3 genotyping | This study |
| FEP3-OX3_chr1 rev | TGTTGTTCTAAATTTTGGGATCTT | FEP3-OX#3 genotyping | This study |
| btsl1-1_LP | TGCTTGGCATAATCCTTCTTG | *btsl1* allele genotyping | (Rodríguez-Celma et al., 2017) |
| btsl1-1_RP | GAACTCTCTTGCTTCCTGAAGC | *btsl1* allele genotyping | (Rodríguez-Celma et al., 2017) |
| btsl2-2_LP | TCGGTTATTCAGGCAAAACAC | *btsl2* allele genotyping | (Rodríguez-Celma et al., 2017) |
| btsl2-2_RP | CCCTTTGTACTCATCAGCAGC | *btsl2* allele genotyping | (Rodríguez-Celma et al., 2017) |
| **RT-qPCR** | | | |
| FEP3_stn fw | GGCCATCAAGAGATTTGACC | *FEP3* RT-qPCR mass standard | This study |
| FEP3_stn rev | TGGAAACCATGTTTGTTCATCT | *FEP3* RT-qPCR mass standard | This study |
| FEP3_qPCR fw | TGCTTCCACCGTGTATGTTG | *FEP3* RT-qPCR | This study |
| FEP3_qPCR rev | CAGGAGCATAATCATAGCCACTG | *FEP3* RT-qPCR | This study |
| BTSL1_stn fw | TCCCTGGATCCTCAGAAGAA | *BTSL1* RT-qPCR mass standard | This study |
| BTSL1_stn rev | CGGCTGGTTCTACAATGATG | *BTSL1* RT-qPCR mass standard | This study |
| BTSL1_qPCR fw | CTCCCCAGTGAAGGCTCTTC | *BTSL1* RT-qPCR | This study |
| BTSL1_qPCR rev | GACCTGCATATCTCCAAGCGA | *BTSL1* RT-qPCR | This study |
| BTSL2_stn fw | ATGAGCCGTTGGATTGCTAC | *BTSL2* RT-qPCR mass standard | This study |
| BTSL2_stn rev | CAAAATCAAATGCTTCCAAAAA | *BTSL2* RT-qPCR mass standard | This study |
| BTSL2_qPCR fw | GCTTGTATGTCGCGACTCAT | *BTSL2* RT-qPCR | This study |
| BTSL2_qPCR rev | AGAGCCTTCACCGGAGAATT | *BTSL2* RT-qPCR | This study |
| BTS_stn fw | AACTTGGATGTTCCCCGTCT | *BTS* RT-qPCR mass standard | This study |
| BTS_stn rev | ATCAACGGGCTTCTTCACAT | *BTS* RT-qPCR mass standard | This study |
| BTS_qPCR fw | CGGGGAAGGACTAGGAATCG | *BTS* RT-qPCR | This study |
| BTS_qPCR rev | CAGCAGATGGGGCAATTTGT | *BTS* RT-qPCR | This study |
| STD-BHLH038-5' | GGAGATAACCTAAATAACGGC | *BHLH38* RT-qPCR mass standard | (Naranjo Arcos, 2017) |
| STD-BHLH038-3' | GGTCCAGATCAGTGTTAGATTCA | *BHLH38* RT-qPCR mass standard | (Naranjo Arcos, 2017) |
| RT 5'bHLH38 | AGCAGCAACCAAAGGCG | *BHLH38* RT-qPCR | (Wang et al., 2007) |
| RT 3'bHLH38 | CCACTTGAAGATGCAAAGTGTAG | *BHLH38* RT-qPCR | (Wang et al., 2007) |
| STD-BHLH039-5' | AACCAAAGCAGCTTCCAAG | BHLH39 RT-qPCR mass standard | (Naranjo Arcos, 2017) |
| STD-BHLH039-3' | CGAAGAGAAAAAGGACGACA | BHLH39 RT-qPCR mass standard | (Naranjo Arcos, 2017) |
| bHLH039-RT 5' | GACGGTTTCTCGAAGCTTG | BHLH39 RT-qPCR | (Wang et al., 2007) |
| RT 3'bHLH39 | GGTGGCTGCTTAACGTAACAT | BHLH39 RT-qPCR | (Wang et al., 2007) |
| STD-FIT-5'(MN) | AAGACATGACCAAAAATGTGTGT | *FIT* RT-qPCR mass standard | (Naranjo Arcos, 2017) |
| STD-FIT-3'(MN) | TGCATCTCCAACAATGGATGC | *FIT* RT-qPCR mass standard | (Naranjo Arcos, 2017) |
| FIT F (g166-187) | CCCTGTTTCATAGACGAGAACC | *FIT* RT-qPCR | (Naranjo Arcos, 2017) |
| RT-FIT-3'(MN) | ATCCTTCATACGCCCTCTCC | *FIT* RT-qPCR | (Bauer, 2016) |
| AtIRT1-temp-5’(898) | TAGCCATTGACTCCATGGC | *IRT1* RT-qPCR mass standard | (Klatte, 2008) |
| AtIRT1-temp-3’(1910) | AGAAAACTATGAATCGTGGGG | *IRT1* RT-qPCR mass standard | (Klatte, 2008) |
| AtIRT1-c-5'new | AAGCTTTGATCACGGTTGG | *IRT1* RT-qPCR | (Wang et al., 2007) |
| AtIRT1-c-3' (1622) | TTAGGTCCCATGAACTCCG | *IRT1* RT-qPCR | (Wang et al., 2007) |
| AtFRO2-temp-5'(3110) | CCATGCTCGATCTTGTCTTG | *FRO2* RT-qPCR mass standard | (Bauer, 2016) |
| AtFRO2-temp-3’(4105) | ATTCCGGAACTTTTGAAAGG | *FRO2* RT-qPCR mass standard | (Bauer, 2016) |
| FRO2-c5-RT5' | CTTGGTCATCTCCGTGAGC | *FRO2* RT-qPCR | (Wang et al., 2007) |
| FRO2-c3-RT3' | AAGATGTTGGAGATGGACGG | *FRO2* RT-qPCR | (Wang et al., 2007) |
| PYE_stn fw | ACCGAAAAGGATCAACAAGG | *PYE* RT-qPCR mass standard | This study |
| PYE _stn rev | CCATCAAGGCCATAACTTCC | *PYE* RT-qPCR mass standard | This study |
| PYE _qPCR fw | GTTCCCAGGACTTCCCATTT | *PYE* RT-qPCR | This study |
| PYE _qPCR rev | GTGTCTGGGGATCAGGTTGT | *PYE* RT-qPCR | This study |
| FRO3_stn fw | AATCAGATCGACCACCTTGC | *FRO3* RT-qPCR mass standard | This study |
| FRO3_stn rev | TTCTTTTGGTGAGAAGATTTTGG | *FRO3* RT-qPCR mass standard | This study |
| FRO3_qPCR fw | ATCGACCACCTTGCTGTTTC | *FRO3* RT-qPCR | This study |
| FRO3_qPCR rev | TTATCCCACTGCCTCCACTC | *FRO3* RT-qPCR | This study |
| NAS4_stn fw | CACTCTCTTCAAGCAGCTCGT | *NAS4* RT-qPCR mass standard | This study |
| NAS4_stn rev | CTGTAGCAAAAACAGCCAACA | *NAS4* RT-qPCR mass standard | This study |
| AtNAS4-RT810-5’ | TGTAATCTCAAGGAAGCTAGGTG | *NAS4* RT-qPCR | (Klatte et al., 2009) |
| AtNAS4-RT947-3’ | GCGAACTCCTCGATAATGC | *NAS4* RT-qPCR | (Schuler, 2011) |
| ILR3_stn fw | TGATGGCTCGGCTGGAAAC | *ILR3* RT-qPCR mass standard | This study |
| ILR3_stn rev | CTAAGAAAGCCGAGAAAGAGAGGAG | *ILR3* RT-qPCR mass standard | This study |
| ILR3_qPCR fw | GCATGTAGAGAGAAGCAGCGAC | *ILR3* RT-qPCR | This study |
| ILR3_qPCR rev | TGCGGACAGCATCAACCAAG | *ILR3* RT-qPCR | This study |
| bHLH104_stn fw | GAATTTGCAGCAGGAGCCAG | *BHLH104* RT-qPCR mass standard | This study |
| bHLH104_stn rev | GCCAAACGGAAGAATCCTAAACC | *BHLH104* RT-qPCR mass standard | This study |
| bHLH104_qPCR fw | GGTTGAGGAGGGAGAAGCTAAATG | *BHLH104* RT-qPCR | This study |
| bHLH104_qPCR rev | ACGGATTGCATCATCGAGTATAGC | *BHLH104* RT-qPCR | This study |
| STD-EF1Balpha2-5' | GCTGCTAAGAAGGACACCAAG | *EF1Balpha* (genomic) RT-qPCR mass standard | (Bauer, 2016) |
| STD-EF1Balpha2-3' | TGTTCTGTCCCTACTGGATCC | *EF1Balpha* (genomic) RT-qPCR mass standard | (Bauer, 2016) |
| EFc-5' | TATGGGATCAAGAAACTCACAAT | *EF1Balpha* RT-qPCR | (Bauer, 2016) |
| EFc-3' | CTGGATGTACTCGTTGTTAGGC | *EF1Balpha* RT-qPCR | (Wang et al., 2007) |
| AtEF-gen-5' (2522) | TCCGAACAATACCAGAACTAC | *EF1Balpha* (genomic) RT-qPCR | (Mai et al., 2016) |
| AtEF-gen-3' (2726) | CCGGGACATATGGAGGTAAG | *EF1Balpha* (genomic) RT-qPCR | (Wang et al., 2007) |
| **Amplification of full-length CDS or deletion constructs for Y3H** | | | |
| FIT-C_AQ fw | AAAGGTCAAAGACAGTTGACTGTATCGCCGGAATTCATGACTCAACCTTTTCGCGGTATC | Cloning of FIT-C in pBRIDGE-GW using AQUA Cloning | This study |
| FIT-C_AQ rev | CGCCCGGAATTAGCTTGGCTGCAGGTCGACGGATCCTCAAGTAAATGACTTGATG | Cloning of FIT-C in pBRIDGE-GW using AQUA Cloning | This study |
| bHLH39_AQ fw | AAAGGTCAAAGACAGTTGACTGTATCGCCGGAATTCATGTGTGCATTAGTACCTC | Cloning of bHLH39 in pBRIDGE-GW using AQUA Cloning | This study |
| bHLH39_AQ rev | CGCCCGGAATTAGCTTGGCTGCAGGTCGACGGATCCTCATATATATGAGTTTCCAC | Cloning of bHLH39 in pBRIDGE-GW using AQUA Cloning | This study |
| BTSL1-C_AQ fw | AAAGGTCAAAGACAGTTGACTGTATCGCCGGAATTCATGCCTAATTACAAGGTTGAAGTTGGC | Cloning of BTSL1-C in pBRIDGE-GW using AQUA Cloning | This study |
| BTSL1-C_AQ rev | CGCCCGGAATTAGCTTGGCTGCAGGTCGACGGATCCCTAAAGGAGCCTTGAGTTGTAG | Cloning of BTSL1-C in pBRIDGE-GW using AQUA Cloning | This study |
